# Using atomistic solution scattering modelling to elucidate the role of the Fc glycans in human IgG4

**DOI:** 10.1371/journal.pone.0300964

**Published:** 2024-04-01

**Authors:** Valentina A. Spiteri, James Doutch, Robert P. Rambo, Jayesh S. Bhatt, Jayesh Gor, Paul A. Dalby, Stephen J. Perkins

**Affiliations:** 1 Division of Biosciences, Department of Structural and Molecular Biology, University College London, London, United Kingdom; 2 ISIS Facility, STFC Rutherford Appleton Laboratory, Harwell Campus, Didcot, Oxfordshire, United Kingdom; 3 Diamond Light Source Ltd., Diamond House, Harwell Science and Innovation Campus, Chilton, Didcot, Oxfordshire, United Kingdom; 4 Department of Biochemical Engineering, University College London, London, United Kingdom; Chang Gung University, TAIWAN

## Abstract

Human immunoglobulin G (IgG) exists as four subclasses IgG1-4, each of which has two Fab subunits joined by two hinges to a Fc subunit. IgG4 has the shortest hinge with 12 residues. The Fc subunit has two glycan chains, but the importance of glycosylation is not fully understood in IgG4. Here, to evaluate the stability and structure of non-glycosylated IgG4, we performed a multidisciplinary structural study of glycosylated and deglycosylated human IgG4 A33 for comparison with our similar study of human IgG1 A33. After deglycosylation, IgG4 was found to be monomeric by analytical ultracentrifugation; its sedimentation coefficient of 6.52 S was reduced by 0.27 S in reflection of its lower mass. X-ray and neutron solution scattering showed that the overall Guinier radius of gyration *R*_*G*_ and its cross-sectional values after deglycosylation were almost unchanged. In the *P(r)* distance distribution curves, the two *M1* and *M2* peaks that monitor the two most common distances within IgG4 were unchanged following deglycosylation. Further insight from Monte Carlo simulations for glycosylated and deglycosylated IgG4 came from 111,382 and 117,135 possible structures respectively. Their comparison to the X-ray and neutron scattering curves identified several hundred best-fit models for both forms of IgG4. Principal component analyses showed that glycosylated and deglycosylated IgG4 exhibited different conformations from each other. Within the constraint of unchanged *R*_*G*_ and *M1-M2* values, the glycosylated IgG4 models showed more restricted Fc conformations compared to deglycosylated IgG4, but no other changes. Kratky plots supported this interpretation of greater disorder upon deglycosylation, also observed in IgG1. Overall, these more variable Fc conformations may demonstrate a generalisable impact of deglycosylation on Fc structures, but with no large conformational changes in IgG4 unlike those seen in IgG1.

## Introduction

**Fig 1:**
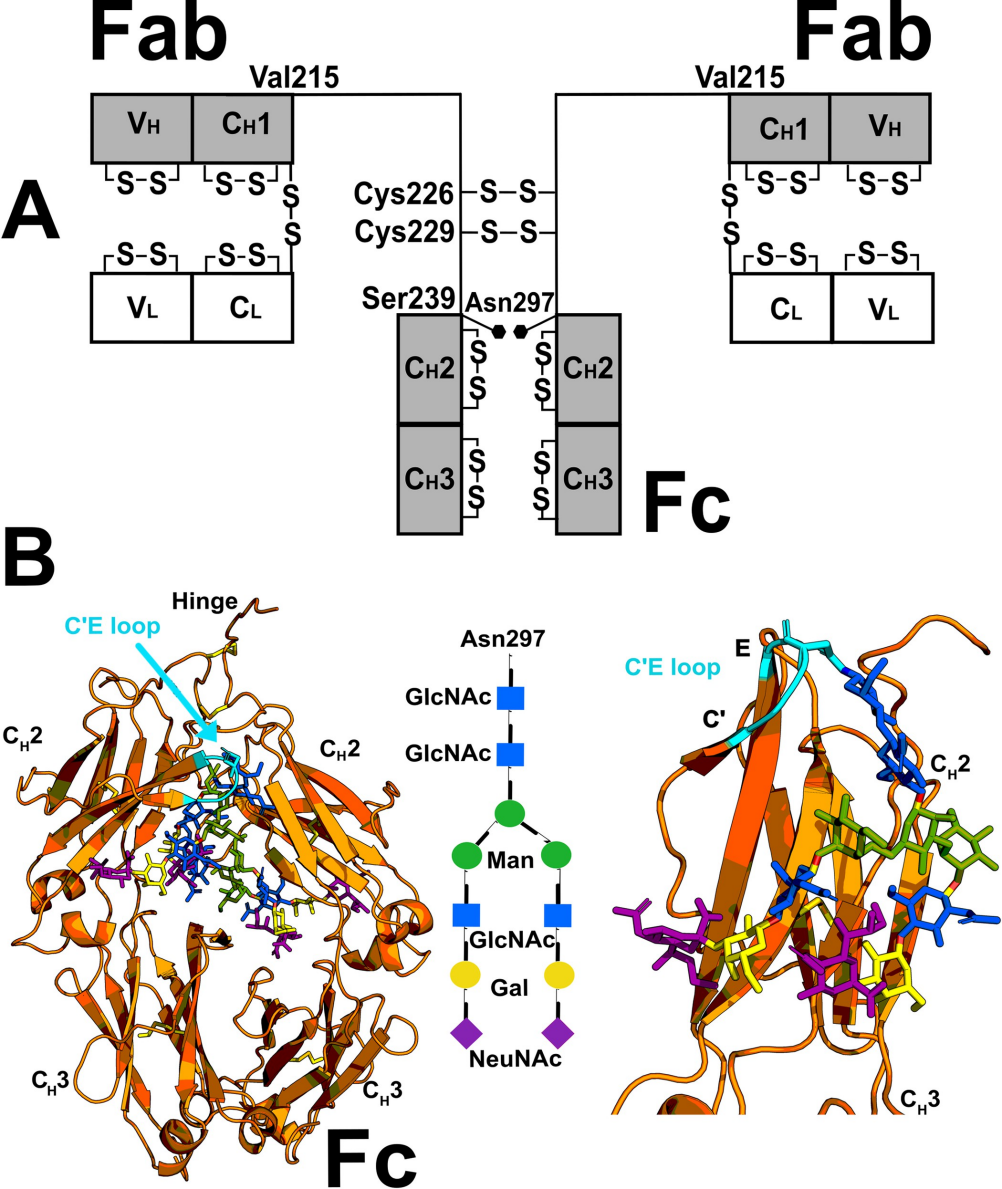
The domains in human IgG4 and their glycosylation. (A) The light chains are constructed of the V_L_ and C_L_ domains and the heavy chains are constructed of the V_H_, C_H_1, C_H_2 and C_H_3 domains. Two Cys-Cys disulphide bridges connect the heavy chains at Cys-226 and Cys-229. An N-linked glycan is located on each C_H_2 domain at Asn-297. The extended 20-residue hinge ^216^ESKYGPPCPPCPAPEFLGGP^235^ connects the Fab and Fc subunits. (B) Left, the glycans of IgG4 Fc are shown as a stick representation (PDB ID: 4C55). The three hinge tripeptides used in the TAMC searches are circled in green. Centre, schematic of the glycan structure (mannose, Man; galactose, Gal; *N*-acetyl glucosamine, GlcNAc; *N*-acetyl neuraminic acid, NeuNAc. Right, a single glycosylated C_H_2 domain, where the glycan residue colours follow those in the central schematic.

Human immunoglobulin G (IgG) is the most abundant antibody in blood plasma/serum and is often exploited as a biotherapeutic due to its high specificity to antigens. The four IgG subclasses are termed IgG1, IgG2, IgG3 and IgG4, which are numbered according to their serum concentrations which are 8.0 mg/ml, 4.0 mg/ml, 0.8 mg/ml and 0.4 mg/ml in that order [1]. IgG is arranged as a characteristic Y-shape, comprised of two Fab subunits that bind with high affinity and specificity to an antigen, together with a single Fc subunit that interacts with Fcγ receptors (FcγRs) (Fig 1*A*). The Fab and Fc subunits are connected by hinges of varying lengths depending on the subclass. Of the four subclasses, IgG4 has the shortest hinge with 12 residues ESKYGPPCPPCP that are connected by two Cys-226 and Cys-229 disulphide bridges (Fig 1*A*) [2]. IgG4 has several interesting characteristics, including its ability to undergo Fab arm exchange, whereby the heavy chains dissociate and reassemble *in vivo* to form bispecific antibodies [3]. Fab arm exchange means that IgG4 can behave as though it is monovalent with two different antigen binding sites and this prevents cross-linking of antibody-antigen complexes, further contributing to its anti-inflammatory nature [4]. Fab arm exchange can be abrogated by a S225P hinge mutation (S1 Fig in S1 File) [3]. In addition, IgG4 is considered an anti-inflammatory antibody due to its inability to activate complement [4], unlike its other IgG counterparts. Compared to IgG1, IgG4 has a reduced binding affinity for pro-inflammatory FcγRs, including FcγRIIIa, which is implicated in antibody-dependent cytotoxicity [5]. These anti-inflammatory characteristics makes IgG4 a desirable therapeutic treatment for pathologies in which inflammation is problematic. Twelve approved IgG4-based antibodies are available on the market [6].

IgG immunoglobulins have a conserved N-linked glycan at Asn-297 of the C_H_2 domain of the Fc subunit, which is important for function (Fig 1*B*). Each glycan is typically formed as a Man_3_GlcNAc_2_ core with two NeuNAc.Gal.GlcNAc appendages [7] (Fig 1), however the composition of the two glycans is chemically heterogenous [8]. The IgG-Fc glycan modulates the binding specificity of FcγRs [9]. The ability to engineer the Fc glycan is of growing interest in biotherapeutics, in which the aim is to modulate IgG4 function by influencing which FcγRs it can bind to. For example, afucosylated IgG4 antibodies are able to elicit a stronger antibody-dependent cytotoxicity response through their binding to FcγRIIIa receptors [10]. Deglycosylated IgG4 was unable to bind to FcγRIIIa, indicating the importance of the glycans for FcγR binding [11]. Structures for IgG4 are essential to understand how it binds to FcγRs. Two detailed crystal structures for full length IgG4 (PDB ID: 5D43 and 6GFE) [12, 13] provide only a single view of the IgG4 structure and not the full conformational space that the Fc and Fab subunits can occupy in solution. Six glycosylated IgG4 Fc crystal structures showed the glycans facing inward within the C_H_2 domains (PDB ID: 4C54, 4C55, 5LG1, 5W5M and 5W5N) [14–16]. One high-resolution structure of a deglycosylated IgG4-Fc (PDB ID: 4D2N) showed that the C_H_2-C_H_2 domain interactions bury in part the C_H_2 surface that would be solvent exposed with the glycan present [17]. Unfortunately, crystal structures for IgG-Fc in complex with the FcγRs are limited to the IgG1 subclass. Therefore, it is currently difficult to deduce the molecular basis for FcγRs binding to IgG4-Fc and how the glycans might be involved in this interaction.

The structural effect of the two Fc glycans on full-length IgG4 is unknown, although we have recently investigated this issue for IgG1 [18], which found that deglycosylated IgG1 was conformationally more flexible and extended than glycosylated IgG1 [18]. Given that IgG1 stability and structure was sensitive to deglycosylation, this makes it challenging to develop non-glycosylated therapies based on IgG1. The advantages of non-glycosylation would be cheaper manufacture in bacterial systems and greater homogeneity of the product. This then raised the question as to whether IgG4 would be a better candidate, so we examined the impact of deglycosylation on IgG4. Here, by following the same procedures as in our IgG1 study [18], we determined atomistic solution structures for IgG4 by using analytical ultracentrifugation (AUC) and small angle scattering by X-rays and neutrons (SAXS, SANS), which were combined with Monte Carlo modelling to determine best-fit structures. X-rays provide data sets using a high positive solute-solvent contrast that highlight the hydrophilic surface regions of IgG4, while neutrons in heavy water buffers provides data sets using a high negative solute-solvent contrast that highlights the buried hydrophobic core of IgG4 [19–21]. The tightly-bound hydration layer on the protein surface is visible by X-rays because its electron density is similar to that of the protein and not to bulk water, and is much less visible by neutrons. The Monte Carlo atomistic modelling of the scattering curves leads to best-fit molecular models [22]. Previously we showed that the Fab subunits in IgG4 restricted access to the Fc subunit to limit the binding of Fc to its FcγRs and C1q ligands [23]. Here, we show that deglycosylation also gave a detectably more mobile Fc structure in IgG4, as for IgG1, with more degrees of freedom in IgG4 to access more conformational space and that the glycans seem to preorder the system. Nonetheless this study revealed a greater stability in the IgG4 structure after deglycosylation compared to IgG1, and accordingly IgG4 shows a better potential as a non-glycosylated therapy than IgG1.

## Materials & methods

### Purification and composition of IgG4

IgG4 A33 (146.9 kDa) was kindly provided by Dr John O’Hara and Dr Bernie Sweeney (Union Chimique Belge (UCB) Pharma Ltd., Slough, Berkshire, UK). Its glycans were removed using peptide:N-glycosidase F (PNGase F) according to the manufacturer’s protocol (35.5 kDa, New England Biolabs, Massachusetts, USA) [24]. 3.7μl PNGase F (1850 activity units) was used with 150 μl of IgG4 (14.0 mg/ml). IgG4 was incubated at 37°C for 1 hour (TP1), 6 hour (TP6) and 10 hour (TP10) time points. Amicon Ultra-0.5 ml centrifugal filters (100 kDa cut-off) were used afterwards to pass the PNGase F through the membrane as well as concentrating the deglycosylated IgG4. Prior to SAXS, SANS and AUC measurements, IgG4 samples were gel filtrated to remove any non-specific aggregates using a Super 6 Increase 10/300 GL column (Cytiva, Amersham, UK), concentrated using Amicon Ultra-15 spin concentrators (100 kDa cut-off), and dialyzed at 4°C into 20 mM L-histidine, 138 mM NaCl, 2.6 mM KCl buffer, pH 6.0. This histidine buffer improved the solution stability of IgG4. The sequence of IgG4 A33 was aligned with those from IgG4 Ser-222 and IgG4 Pro-222 [23], IgG4 Fab from B72.3 (PDB ID: 1BBJ) [25], and IgG4 Fc (PDB ID: 4C55) [14] (S1 Fig in S1 File). The Asn-297 glycan was approximated as a Man_3_GlcNAc_2_ core and two NeuNAc.Gal.GlcNAc antennae [7]. The sequence gave solution parameters that were summarised in Table 1 [18]. The completeness of deglycosylation was verified by Superose 6 gel filtration, SDS-PAGE, LC-MS mass spectrometry [26], and AUC (below). The buffer density was measured on an Anton Paar DMA 5000 density meter at 20°C to be 1.00578 g/ml for light water and 1.11106 g/ml for heavy water. Buffer viscosities were measured on an Anton Paar AMVn Automated microviscometer at 20°C to be 0.010190 and 0.01384 poise for light and heavy water respectively.

**Table 1:** Solution parameters for human IgG4 A33.

Parameter	Glycosylated IgG4	Deglycosylated IgG4
Calculated mass (kDa)	148.1	143.7
Unhydrated volume (nm3)	190.7	186.0
Hydrated volume (nm3)	251.2	244.6
Partial specific volume $\bar{v}$ (ml/g)	0.730	0.732
Absorption coefficient (1%, 1 cm)	14.0	14.5

### Sedimentation velocity data and analysis of IgG4

AUC data were obtained at 20°C on two Beckman XL-I instruments with AnTi50 rotors (Beckman Coulter, High Wycombe, UK). Rotor speeds of 30,000 and 40,000 rpm were used with two-sector cells with column heights of 12 mm for ~6 hours. The protein concentrations were 0.96–3.76 mg/ml (glycosylated), 0.81–3.05 mg/ml (TP1), 0.88–4.40 mg/ml (TP6) and 0.65–2.44 mg/ml (TP10). SEDFIT (version 15.01b) analyses employed direct boundary Lamm fits of up to 900 scans [27, 28] to give size-distribution analyses *c(s)* in which all species were taken to have the same frictional ratio *f/f*_*0*_. *A fixed resolution of 200 was used*, *and the c(s)* fits were optimised by floating *f/f*_*0*_ and the baseline until the visual appearance of the fits and the overall root mean square deviations were satisfactory. *C(s)* integrations gave the percentage of oligomers in the analysis. Values were corrected to *s*_*20*,*w*_ by:

$$s_{20,w}=s_{T,B}\left(\frac{\eta_{T,B}}{\eta_{20,w}}\right)\frac{(1-\bar{v}\rho {)}_{20,w}}{(1-\bar{v}\rho {)}_{T,B}}$$

where *s* is the observed sedimentation coefficient, *T*,*B* refers to the temperature of the buffer, *20*,*w* refers to water at 20°C, *η* is the solvent viscosity *ρ* is the solvent density, and $\bar{v}$ is the IgG4 partial specific volume.

### X-ray and neutron scattering data and analyses for IgG4

X-ray scattering data was obtained during one beam session on Instrument B21 at the Diamond Light Source, operating with a ring energy of 3 GeV, and a beamline operational energy of 12.4 keV [29]. A PILATUS 2M detector with a resolution of 1475 × 1679 pixels (pixel size of 172 × 172 μm) was used with a sample-to-detector distance of 4.01 m giving a *Q* range from 0.04 nm^-1^ to 4 nm^-1^ (where *Q* = 4 π sin θ / λ; 2θ = scattering angle; λ = wavelength). The IgG4 (1.33–4.03 mg/ml), TP1 (1.63–4.70 mg/ml), TP6 (1.06–3.10 mg/ml) and TP10 (0.85–3.07 mg/ml) samples in light water were loaded in an EMBL Arinax sample holder [30]. An automatic sampler injected 30 μl of sample from the 92-well plate into a temperature-controlled quartz capillary of diameter 1.5 mm. Data acquisitions of 30 frames with a 1 second exposure time each were used, together with checks to confirm no radiation damage. ScÅtter (version 3.0) was used for buffer subtraction, data reduction and averaging the 30 frames [31].

Neutron scattering data on the IgG4 (1.76–6.05 mg/ml), TP1 (0.71–1.93 mg/ml), TP6 (2.38 mg/ml) and TP10 (5.62 mg/ml) samples in heavy water were obtained in two sessions on Instrument SANS2D at the ISIS pulsed neutron source [32]. Proton beam currents of ~40 μA gave a pulsed neutron beam, from which SANS2D data were recorded with 4 m collimation, 4 m sample-detector distance, a 12 mm sample aperture, and a time-of-flight wavelength range of 0.175–1.65 nm. Data acquisition using a two-dimensional ^3^He detector with 512 × 512 pixels of 7.5 × 75 mm^2^ in size gave a *Q* range from 0.05 nm^-1^ to 4 nm^-1^. Samples of volume 1 ml were measured in circular banjo cells of 2 mm path length for 1–7 h in a thermostatted sample rack at 20°C. The MANTID data reduction [33] included corrections for the *Q* resolution, i.e. beam divergence effects and smearing from the shape and size of the slits, as well as the wavelength overlap in each pulse. SASview software showed that the Guinier analyses (below) were almost unaffected if the smearing was turned on or off.

Guinier scattering analyses gave the radius of gyration *R*_*G*,_ the cross-sectional radius of gyration *R*_*XS*_, and the molecular mass. In a given solute-solvent contrast, the radius of gyration *R*_*G*_ monitors structural elongation if the internal inhomogeneity of scattering densities within the protein has no effect. Guinier analyses at low *Q* gave the *R*_*G*_ value and the forward scattering at zero angle *I(0)* [34]:

$$\ln\;I(Q)=\ln\;I(0)-\frac{{R_{G}}^{2}Q^{2}}{3}$$

For antibodies, this expression is valid in a *Q*.*R*_*G*_ range up to 1.5 [22, 35, 36]. If the structure is elongated, the mean radius of gyration of the cross-sectional structure *R*_*XS*_ and the mean cross-sectional intensity at zero angle [I(Q)Q]_Q→0_ is obtained from [37, 38]:

$$\ln[I(Q)Q]={\left[I(Q)Q\right]}_{Q⟶0}-\frac{{R_{XS}}^{2}Q^{2}}{2}$$

The cross-sectional plot for antibodies exhibits two regions, a steeper innermost one and a flatter outermost one [37, 38], being denoted by *R*_*XS-1*_ and *R*_*XS-2*_ respectively. *R*_*XS-1*_ represents the averaged spatial separation of the Fab and Fc subunits, while *R*_*XS-2*_ represents the averaged cross-section of the two Fab and one Fc subunits. The *R*_*G*_ and *R*_*XS*_ analyses were performed using SCT (S1 Table in S1 File) [39]. The *Q* ranges for *R*_*G*_, *R*_*XS-1*_ and *R*_*XS-2*_ were 0.10–0.22 nm^-1^, 0.29–0.52 nm^-1^, and 0.66–1.05 nm^-1^, respectively [22, 37, 38]. Indirect transformation of the scattering data *I(Q)* which gives the distance distribution function *P(r)* was carried out using GNOM (version 4.6) [40, 41].


$$P(r)=\frac{1}{{2\pi }^{2}}\int_{0}^{\infty }I(Q)Qr\;\sin\left(Qr\right)dQ$$

*P(r)* yields the maximum dimension of the macromolecule *L* and its most commonly occurring distance vector *M* in real space. The X-ray *P(r)* analysis utilized up to 755 data points in the *Q* range between 0.032 and 1.50 nm^-1^. The neutron *P(r)* analysis utilized up to 155 *I(Q)* data points in the *Q* range between 0.055 and 1.60 nm^-1^.

### Atomistic modelling of IgG4

To create the initial structure for IgG4 A33, the A33 sequence provided by UCB Pharma. was aligned with the IgG4 Ser and IgG4 Pro sequences using Clustal Omega software [22, 42] (S1 Fig in S1 File). The Fab structure (Fig 1) was taken from the IgG4 b72.3 crystal structure (PDB ID: 1BBJ) [35] and the Fc structure was taken from the serum-derived IgG4 Fc crystal structure (PDB ID: 4C55) [14]. The A33 Fab sequence was substituted into the IgG4 b72,3 Fab structure using Modeller (Version 9.19) [43]. The peptide ^216^ESKYGPPCPPCPAPEFLGGP^235^ with 20 residues that included the 12-residue IgG4 hinge peptide ^216^ESKYGPPCPPCP^227^ was constructed using a PyMOL script build_seq (PyMOL Script Repository, Queen’s University, Ontario, Canada). The two N-linked glycans at Asn-297 were approximated as complex-type biantennary oligosaccharides with a Man_3_GlcNAc_2_ core and two NeuNAc.Gal.GlcNAc antennae [7]. The glycan template was taken from the https://github.com/dww100 repository and energy minimized using NAMD for 1 nanosecond [44]. To add the glycan to the Fc subunit, its C1 atom in the first GlcNAc residue was positioned to within 0.14 nm of the Asn-297 N sidechain atom. Discovery Studio (Dassault Systèmes BIOVIA, San Diego) created the “CONECT” record for the glycosidic bond. The CHARMM force field parameters and protein structure file (PSF), including those for the disulphide bridges and glycans were generated using the CHARMM-Gui GlycanReader tool [45–47] in order to be compatible with the CHARMM36 forcefield [48–52]. The full IgG4 structure with and without glycans was then energy minimised using NAMD version 2.9 with the CHARMM36 forcefield.

To generate trial IgG4 structures, the initial IgG4 structure was renumbered and renamed to satisfy the format for the Torsion Angle Monte Carlo (TAMC) module in SASSIE-web [53]. The IgG4 residue numbering was changed to be continuous for one segment corresponding to the first Fab subunit, its hinge and the Fc subunit, and the other segment to only the second Fab subunit and the hinge connected to this. Physically realistic IgG4 conformations were created using TAMC in SASSIE-web [53]. The linker regions that were varied to create IgG4 conformers corresponded to six residues ^216^ESK^218^ and ^228^APE^230^ on one hinge of IgG4, and three residues ^216^ESK^218^ on the other hinge (green circles, Fig 1*B*, green text, S1*E* Fig in S1 File). These tripeptides corresponded to surface-accessible structures outside the structurally-defined Fab and Fc subunits and the disulphide-linked hinge core. The rest of IgG4 was held rigid. Making both ^216^ESK^218^ peptides flexible rendered both Fab subunits mobile, and making ^231^APE^230^ flexible made the Fc subunit mobile. For each of these nine linker residues, the backbone phi (φ) and psi (ψ) torsion angles were varied in 15° steps, these 15° steps being found to provide enough sampling to access a wide enough range of conformers for analysis. For glycosylated IgG4, 800,000 moves were attempted of which 111,382 were accepted as sterically acceptable. For deglycosylated IgG4, 600,000 moves were attempted, of which 117,135 models were acceptable.

For each of the 111,382 and 117,135 models, the scattering curve *I(Q)* was calculated using the SasCalc module in SASSIE-web using an all-atom expression in which the orientations of the *Q* vectors are taken from a quasi-uniform spherical grid generated by the golden ratio [54]. X-ray modelling would require the explicit addition of a monolayer of water molecules to the protein surface before calculating *I(Q)*. As this would require much computational effort, as well as only affecting the scattering curve at larger *Q* values [54], the hydration shell was not considered here for X-rays, and was not required for neutrons. The modelled and experimental scattering curves extrapolated to zero concentration were compared using the *R*-factor module in SASSIE-web. The *R*-factor is the difference between the modelled curve *I*_*Model*_*(Q*_*i*_*)* and the interpolated experimental curves *I*_*Expt*_*(Q*_*i*_*)*, this function being analogous to that used in protein crystallography:

$$R=\frac{\sum \left\|\left\|I_{Expt}(Qi)\|-\eta \|I_{Model}(Qi)\right\|\right\|}{\sum \left\|I_{Expt}(Qi)\right\|}\times 100$$

where *Q*_*i*_ is the *Q* value of the *i*^th^ data point, *I*_*Expt*_*(Q*_*i*_*)* is the experimental scattering intensity, *I*_*Model*_ *(Q*_*i*_*)* is the theoretical modelled scattering intensity, and *η* is a scaling factor used to match the theoretical curve to the experimental *I(0)* [34]. Lower *R*-factor values represent better fits [39]. The scattering curves were normalised by dividing the *I(Q)* values by *I(0)*. The experimental scattering curves were interpolated to zero *Q*. Interpolation makes the *Q* spacing uniform between the data points, and extrapolation extends the full *I(Q)* curve to zero *Q*. The resulting 680 and 72 *I(Q)* values in the *Q* range of 0.0–1.5 nm^-1^ were utilised for the X-ray and neutron curve fits respectively, and defined the *Q* spacing for the SasCalc and the *R*-factor values. To evaluate the fits, χ^2^ analyses were not possible because this requires the data points to have errors associated with them, which were not available. For the neutron curve fits, no correction was required for a flat incoherent background because the IgG4 concentrations were relatively low and the dialyses had sufficiently reduced the proton content in the buffers. The 111,382 glycosylated and 117,135 deglycosylated models gave an *R*-factor vs. *R*_*G*_ distribution that encompassed the experimentally deduced *R*_*G*_ value. This *R*-factor analysis was repeated for four experimental X-ray scattering curves at different concentrations for each of glycosylated and deglycosylated IgG4 (S2 Table in S1 File). The same analysis was repeated for two neutron scattering curves at different concentrations, for each of glycosylated and deglycosylated IgG4 (S3 Table in S1 File). For each concentration, the best-fit 100 models with the smallest *R*-factors were accepted.

Principal component analysis (PCA) provided by the Bio3d package in R [55] identified the main types of best-fit IgG4 conformations found in the 800 best-fit glycosylated and deglycosylated models from eight X-ray scattering fits (S2 Table in S1 File). Separately, PCA was applied to the 400 best-fit neutron models. To remove any bias in the PCA clustering of coordinate sets, the glycans were removed from the best-fit glycosylated models prior to generating the PCA. The mid-point structure for each PCA group was identified using a centroid model computed using R. The 100 best-fit models for glycosylated and deglycosylated IgG4 at 4.03 mg/ml and 3.07 mg/ml respectively, including the best fitting models are available in Supplemental Materials. The best-fit glycosylated and deglycosylated IgG4 structures were deposited in the SASDBD database (https://www.sasbdb.org/) with reference codes SASDLX2 and SADLY2.

In order to model AUC parameters the theoretical *s*_*20*,*w*_ values were generated for the best-fit 800 and 400 glycosylated and deglycosylated IgG4 models using HullRad [56]. Hullrad included glycan residues, however there were inconsistencies in the PDB nomenclature for glycans. The nomenclature in the Hullrad script was thus modified to ensure that the IgG4 glycosylation was correctly incorporated in the *s*_*20*,*w*_ calculation.

## Results

### Purification of glycosylated and deglycosylated IgG4

**Fig 2:**
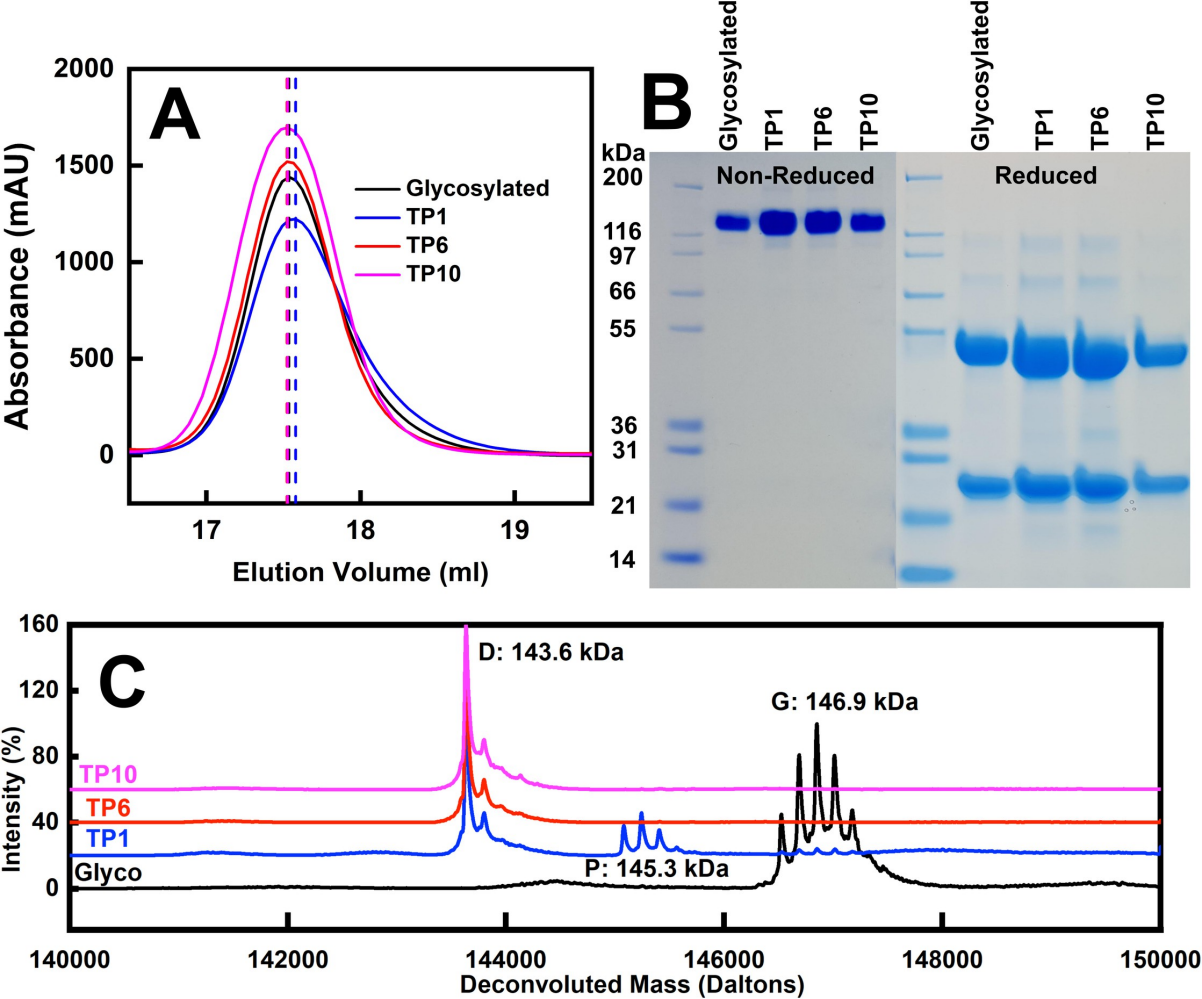
Purification and SDS-PAGE of the IgG4 samples. (A) Elution peaks are shown from a Superose 6 Increase 10/300 gel filtration column for the four IgG4 samples (glycosylated, black; TP1, blue; TP6, red; and TP10, magenta). The peak positions are indicated by dashed vertical lines. (B) Molecular weight markers are denoted in kDa (lanes 1 and 6). SDS-PAGE of non-reduced glycosylated IgG4, TP1, TP6 and TP10 are shown after gel filtration (lanes 2–5). SDS-PAGE of reduced glycosylated IgG4, TP1, TP6 and TP10 are shown after gel filtration (lanes 7–10). (C) Mass spectra are shown for glycosylated and deglycosylated IgG4, to follow the same colour scheme as in (*A*). Glycosylated species are denoted by G, partially glycosylated species are denoted by P, and fully deglycosylated species are denoted by D.

The deglycosylation of human IgG4 A33 employed PNGase F digests (Materials and Methods). The purity of IgG4 was established by SDS-PAGE, gel filtration, and mass spectrometry. At time points of one hour, six hours and ten hours following the start of deglycosylation (termed TP1, TP6 and TP10), the IgG4-digested products were eluted from the gel filtration column slightly prior to glycosylated IgG4 (Fig 2*A*). The proteins eluted as major symmetrical peaks at 17.54 ml, 17.58 ml, 17.52 ml and 17.53 ml for glycosylated IgG4 and the TP1, TP6 and TP10 time points in that order (Fig 2*A*). This procedure was intended to result in monodisperse IgG4 samples that were aggregate-free prior to AUC, SAXS and SANS experiments. SDS-PAGE analyses showed that the four IgG4 preparations gave single bands between 200 and 116 kDa on non-reducing 4–12% Bis Tris NuPage gel, in agreement with the expected ~147 kDa size for IgG4 (Fig 2*B*). Reducing SDS-PAGE gave two bands that were assigned to the light chain (mass of 31–21 kDa) and the heavy chain (mass of ~55 kDa) (Fig 2*B*), in agreement with the masses from its known sequence. Traces of minor bands were visible in the reduced gel that were attributed to partially reduced forms; the mass spectra and AUC results (below) showed that these contaminants were insignificant. Deglycosylation was verified by mass spectrometry in which the molecular mass decreased upon glycan removal (Fig 2*C*). A partially deglycosylated IgG4 denoted as P was observed at TP1. Only a single major peak was seen at 143,636 Da, which showed that deglycosylation was complete at the TP6 and TP10 time points.

### AUC of IgG4 before and after deglycosylation

**Fig 3:**
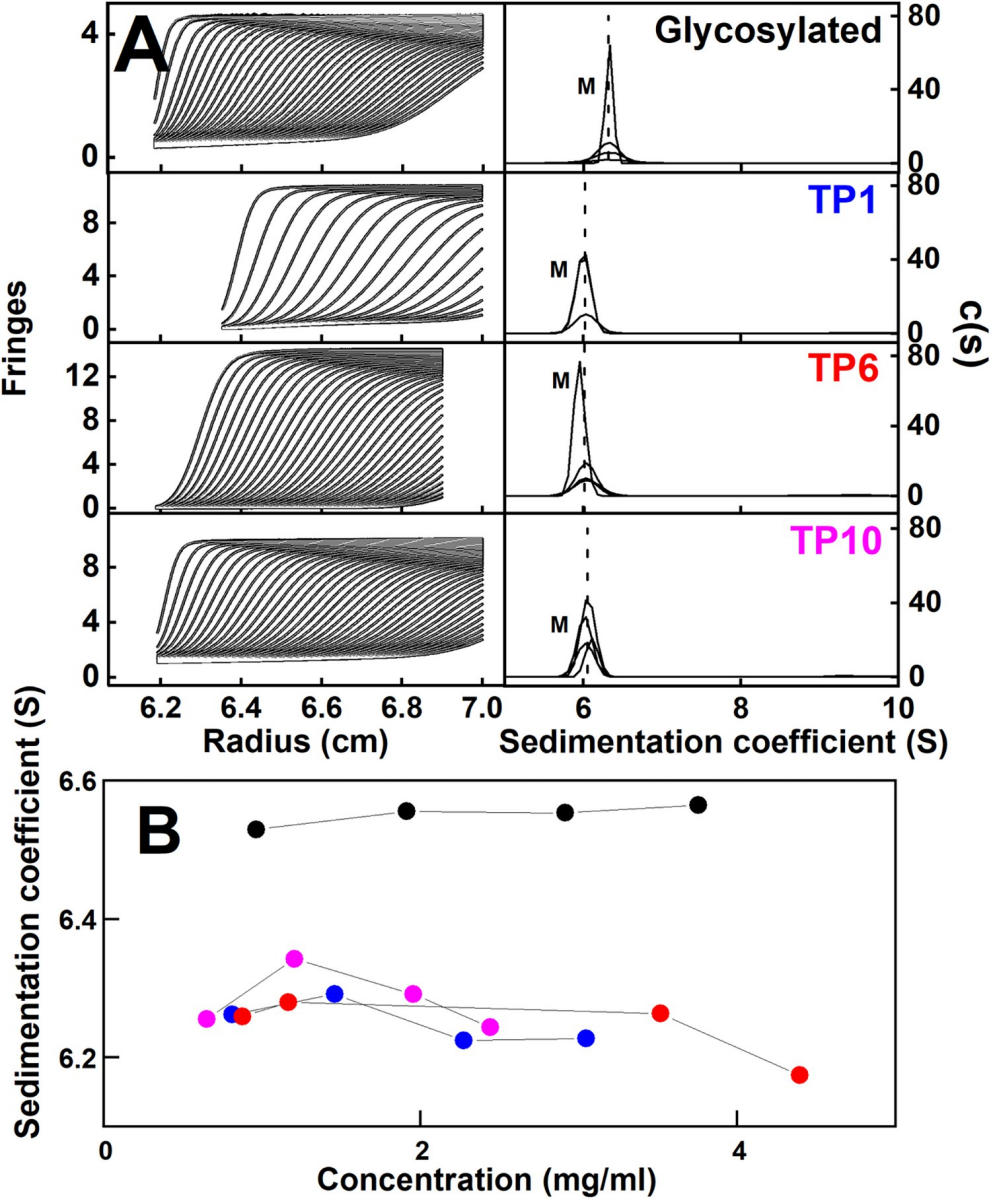
AUC sedimentation velocity analyses of IgG4. (A) Representative experimental AUC sedimentation boundaries are shown for the four IgG4 samples. The TP1 (blue), TP6 (red) and TP10 (magenta) digest timepoints are shown. On the left, the IgG4 concentrations were 2.92 mg/ml (glycosylated), 3.05 mg/ml (TP1), 4.40 mg/ml (TP6) and 2.61 mg/ml (TP10). Shown are 31–66 boundaries (black outlines) from totals of up to 900 scans recorded at 30,000 rpm and 20°C. The fitted SEDFIT curves are shown as white lines. Concentration series (listed in Methods) of each set of four peaks in each *c(s)* analysis revealed monomeric peaks (M) at the *s*^*0*^_*20*,*w*_ values reported in Table 2. (B) The monomer *s*^*0*^_*20*,*w*_ values are shown as a function of IgG4 concentration for glycosylated IgG4 (●), and the IgG4 TP1 (•), TP6 (•) and TP10 (•) time points.

The mass and shape of glycosylated IgG4 and the TP1, TP6 and TP10 digest time points were investigated using AUC sedimentation velocity. Use of the four deglycosylation timepoints provided a useful check of self-consistency between the four samples, all in histidine buffer. The SEDFIT analyses were based on fits of up to 900 scans, and showed good visual agreement between the experimental and fitted boundary scans (Fig 3*A*). The size distribution analyses *c(s)* showed monomer peaks (Table 2; Fig 3*A*). The 6.52 S value for glycosylated IgG4 agreed with those previously of 6.44 S, 6.80 S and 6.60 S for IgG4 (Ser^222^) [57], IgG4 (Ser^222^) and IgG4 (Pro^222^) respectively [23]. No IgG4 dimers were seen as extra peaks at higher *s*_*20*,*w*_ values. The *c(s)* analyses gave the IgG4 masses to be 146–149 kDa (Table 2). These values were comparable with the sequence-calculated masses of 148.1 kDa and 143.1 kDa for glycosylated and deglycosylated IgG4 respectively. A reduction of 5.0 kDa in the mass would lead to a reduction of 0.15 S in the *s*_*20*,*w*_ value according to the Svedberg equation of sedimentation. If the frictional ratio remains constant, the *s*_*20*,*w*_ values should scale with the (mass)^2/3^. The reduction assumed that the IgG4 conformation was unchanged after deglycosylation, and confirmed that the deglycosylation was complete at TP10. In earlier work, minor dimer peaks were visible at ~9 S for IgG4 (Ser^222^) and (Pro^222^) [23]. Here, no dimers were visible for IgG4 A33 (Fig 3*A*), and this was attributed to the use of histidine buffer and not phosphate buffer saline as previous.

**Table 2:** Sedimentation parameters for human IgG4 A33.

Sample	Averaged monomer *s*^*0*^_*20*,*w*_ value (S) ^a^	Svedberg-derived mass (kDa)
Glycosylated IgG4	6.52 ± 0.01	146
IgG4 digest timepoint TP1	6.30 ± 0.01	146
IgG4 digest timepoint TP6	6.29 ± 0.02	148
IgG4 digest timepoint TP10	6.31 ± 0.07	149

^a^ The standard error of the mean are calculated from the values determined in Fig 3.

### Solution scattering of IgG4 before and after deglycosylation

The structures of glycosylated IgG4 and its TP1, TP6 and TP10 forms were investigated by X-ray and neutron solution scattering. From this, the radii of gyration *R*_*G*_ and *R*_*XS*-1_/*R*_*XS-2*_ values monitored the overall IgG4 structure and its cross-sectional structures respectively. X-rays revealed the same solution structure with a visible hydration shell, while neutrons showed a much reduced hydration shell [18–20]. The use of four timepoints provided a key check of self-consistency, when the TP6 and TP10 timepoints reflect complete deglycosylation compared to the starting glycosylated form and the TP1 timepoint should be intermediate between these two states. Furthermore, the use of IgG4 at 0.85–4.03 mg/ml assessed any concentration dependences and time frame analyses ensured that any radiation damage effects were absent.

**Fig 4:**
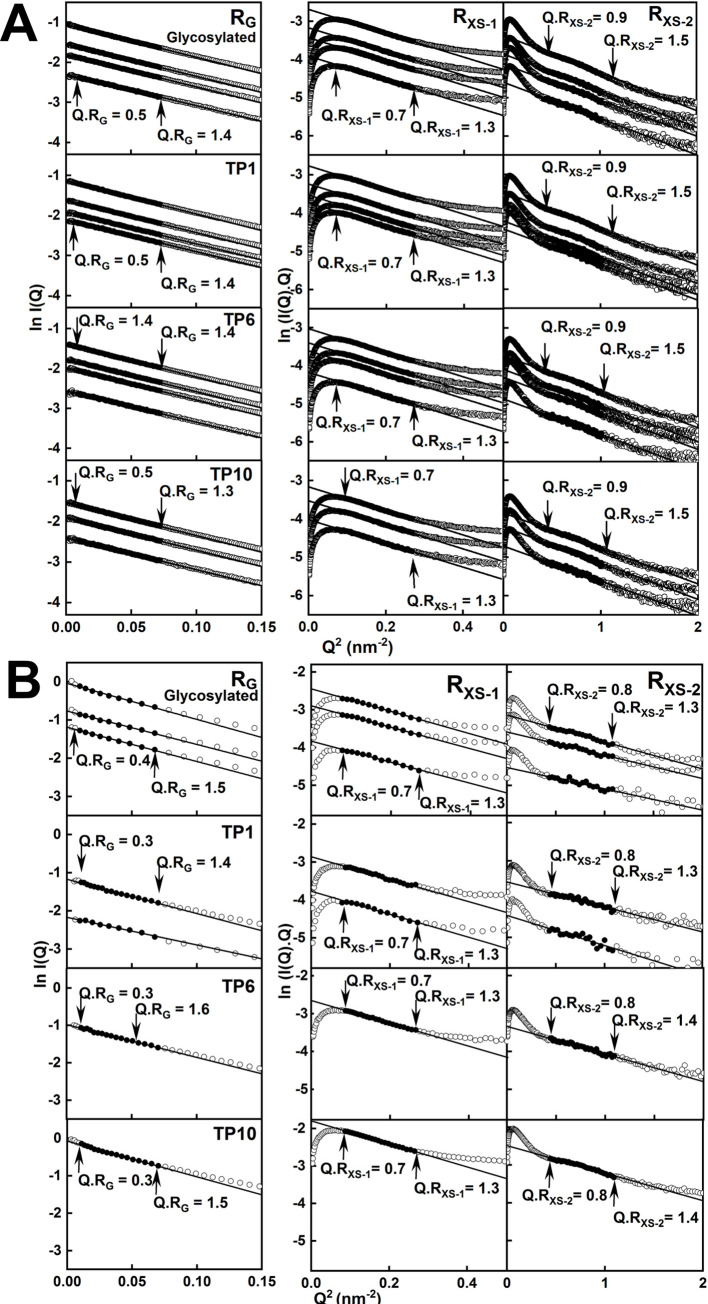
Scattering Guinier analyses for IgG4. (A) The SAXS curves for glycosylated IgG4 and the TP1, TP6 and TP10 samples at 0.85–4.70 mg/ml concentrations. The *Q*.*R*_*G*_ and *Q*.*R*_*XS*_ fit ranges used to determine the *R*_*G*_ and *R*_*XS*_ values are denoted by the filled circles between the vertical arrows. The *Q* ranges used for the *R*_*G*_, *R*_*XS-1*_ and *R*_*XS-2*_ values were 0.10–0.27 nm^-1^, 0.29–0.52 nm^-1^ and 0.66–1.05 nm^-1^ respectively. (B) The corresponding SANS curves for the four samples at 0.71–6.05 mg/ml concentrations. The *Q* ranges used for the *R*_*G*_, *R*_*XS-1*_ and *R*_*XS-2*_ values were 0.07–0.27 nm^-1^, 0.28–0.52 nm^-1^ and 0.66–1.05 nm^-1^ respectively.

**Fig 5:**
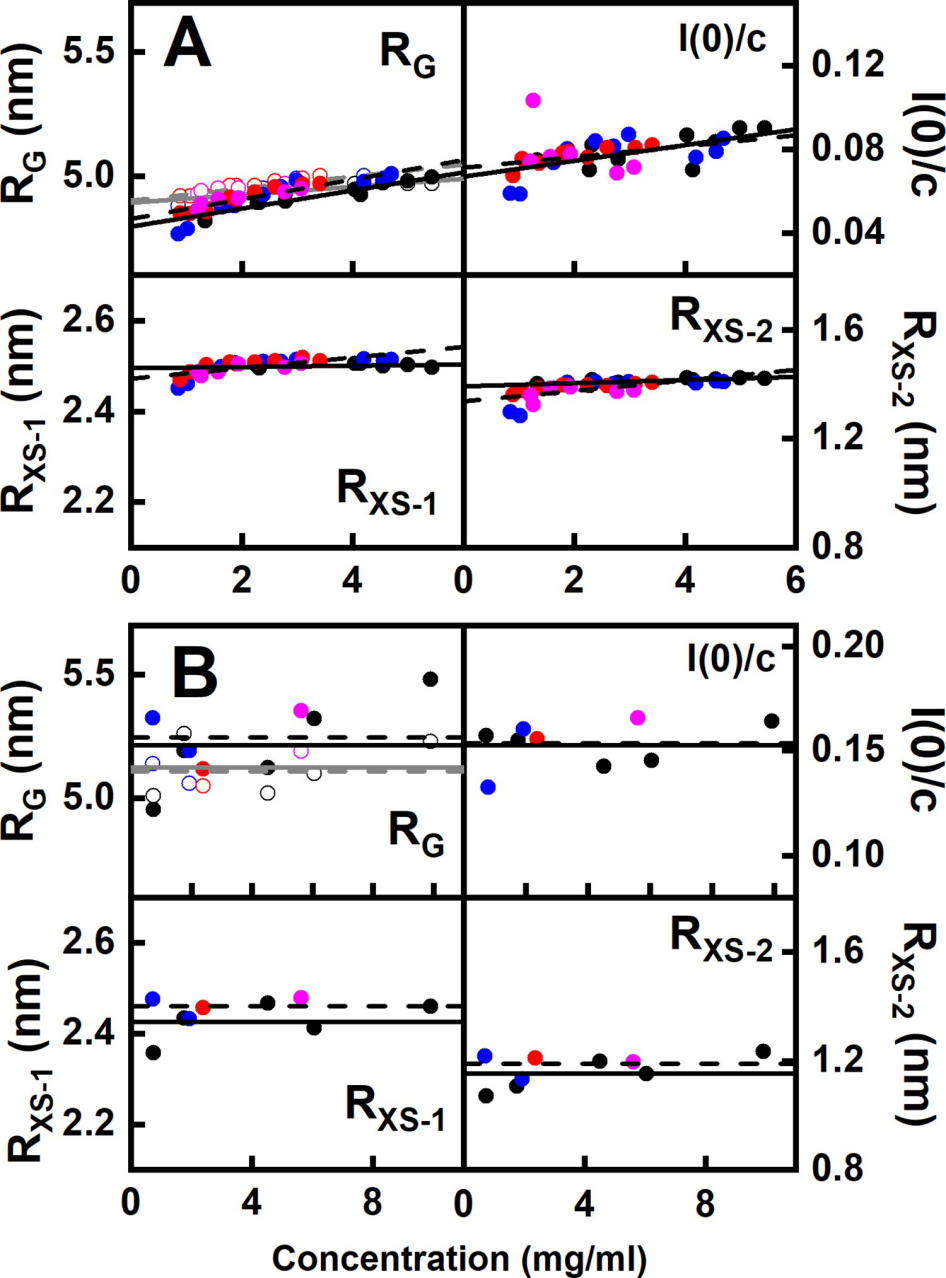
Concentration dependence of the Guinier parameters. In the *R*_*G*_ panels, the filled symbols correspond to the Guinier values and the open symbols correspond to the *P(r)* values. The colours denote the glycosylated IgG4 (black), and the deglycosylated TP1 (blue), TP6 (red) and TP10 (magenta) timepoints. (*A*) The *R*_*G*_, *I(0)/c*, *R*_*XS-1*_ and *R*_*XS-2*_ values from SAXS for the glycosylated (●, ○) and the TP1 (•, ○), TP6 (•, ○) and TP10 (•, ○) samples. The linear regression fits of glycosylated IgG4 are shown by solid lines, and the fits for deglycosylated IgG4 are shown by dashed lines. (*B*) The *R*_*G*_, *I(0)/c*, *R*_*XS-1*_ and *R*_*XS-2*_ values from SANS for the glycosylated and the (TP1, TP6 and TP10) samples. Each corresponds to a single measurement in ^2^H_2_O histidine buffer. The solid and dashed lines show the mean values for glycosylated and deglycosylated IgG4 respectively.

The X-ray Guinier analyses reported three linear regions in the *I(Q)* curves, this being characteristic for immunoglobulins [23, 37, 38]. The *R*_*G*_, *R*_*XS-1*_ and *R*_*XS-2*_ values were determined in satisfactory *Q*.*R*_*G*_ and *Q*.*R*_*XS*_ limits of 0.5–1.4, 0.7–1.3 and 0.9–1.5 in that order (Fig 4*A*). A small concentration dependence in the *R*_*G*_ and *I(0)/c* values suggested some weak protein-protein association between separate IgG4 molecules (Fig 5*A*; S1 Table in S1 File). This concentration dependence was weaker in the *R*_*G*_ values derived from the *P(r)* curves. However IgG4 oligomer formation was ruled out by the absence of dimer peaks or similar in the AUC analyses (Fig 3*A*). The X-ray *R*_*G*_ values at zero concentration for glycosylated IgG4 and TP1, TP6 and TP10 were similar at 4.92 ± 0.19 nm, 4.91 ± 0.27 nm, 4.91 ± 0.17 nm and 4.91 ± 0.13 nm in that order. Those for IgG4 A33 agreed with earlier *R*_*G*_ determinations of 4.82–5.07 nm for monoclonal IgG4 Ser^222^ and IgG4 Pro^222^ antibodies [23]. The *R*_*XS-1*_ values (Fig 5*A*) monitor the IgG4 cross-sectional structure. Their values at zero concentration were unchanged from 2.50 ± 0.01 nm to 2.49 ± 0.10 nm in the four samples, indicating little change in the relative orientation of the Fab and Fc subunits. The *R*_*XS-2*_ values from the individual curves for glycosylated IgG4 and TP1, TP6 and TP10 decreased slightly from 1.41 ± 0.04 nm to 1.38 ± 0.15 nm, 1.39 ± 0.05 nm and 1.37 ± 0.10 nm respectively. Even though the errors were large, the small reduction implied that the Fc subunit became more compact after deglycosylation.

The neutron Guinier data sets for the four IgG4 samples in 100% ^2^H_2_O buffer were analysed in a similar concentration range of 0.71–9.92 mg/ml. Linear Guinier fits were seen for the *R*_*G*_, *R*_*XS-1*_ and *R*_*XS-2*_ values (Fig 4*B*). The mean neutron *R*_*G*_ values for the four samples were 5.21 ± 0.09 nm, 5.26 ± 0.07 nm, 5.10 ± 0.02 nm and 5.35 nm (only one measurement was acquired for the last time point) in that order. This time, no concentration dependence in the neutron *R*_*G*_, *I(0)/c*, *R*_*XS-1*_ and *R*_*XS-2*_ Guinier values was observed, although the reduced number of data points limited the precision of the data sets. The lack of concentration dependence by neutrons and also by AUC suggests that the weak concentration-dependent association noted above may have been induced by X-rays. Fewer concentrations were used for neutrons compared to X-rays, given that the neutron data collection was longer (minimum of one hour compared to one minute for X-rays) and involved larger volumes (800 μl compared to 25 μl for X-rays). The four mean *R*_*XS-1*_ values were comparable at 2.43 ± 0.02 nm, 2.46 ± 0.02 nm, 2.48 ± 0.02 nm and 2.48 nm in that order. The four mean *R*_*XS-2*_ values were comparable at 1.15 ± 0.03 nm, 1.18 ± 0.04 nm, 1.23 ± 0.02 nm and 1.20 nm in that order. The neutron values were lower than the X-ray values for reason of the high negative contrast used for neutrons.

**Fig 6:**
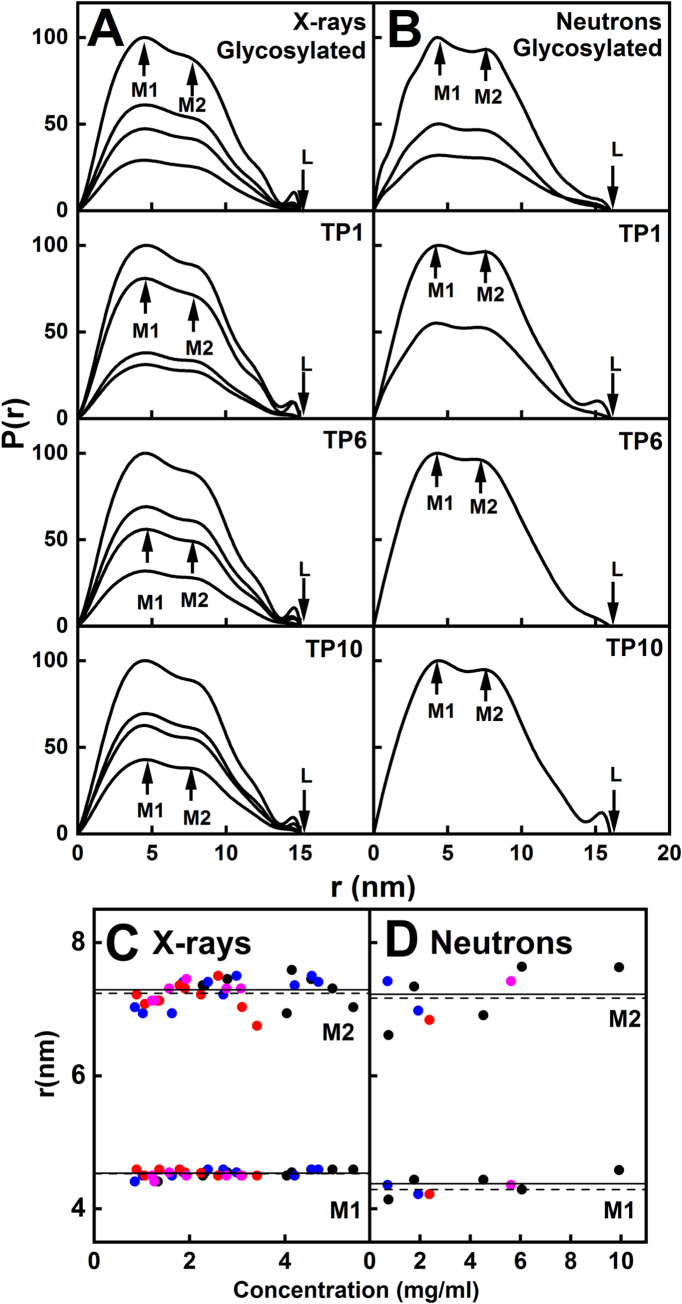
SAXS and SANS distance distribution analyses *P(r)* for IgG4. The concentration series of *P(r)* curves correspond to the SAXS and SANS *I(Q)* curves shown in Fig 4. The colours denote glycosylated IgG4 (black), and the TP1 (blue), TP6 (red) and TP10 (magenta) timepoints. (*A*,*B*) The *M1* and *M2* peak maxima and the maximum length *L* are indicated by arrows. The *P(r)* curves for glycosylated and deglycosylated (TP1, TP6 and TP10) IgG4 are shown at concentrations of 0.85–4.70 mg/ml. (*B*) The corresponding *P(r)* curves for the SANS curves for IgG4 0.71–6.05 mg/ml. (*C*,*D*) The *M1* and *M2* concentration dependences for glycosylated (●) and deglycosylated TP1 (•), TP6 (•) and TP10 (•) IgG4 are shown. The fitted lines are the mean values for glycosylated IgG4 (solid line), and the average of TP1, TP6 and TP10 (dashed lines).

The distance distribution function *P(r)* provided structural data in real space for IgG4. The *P(r)* analyses gave similar *R*_*G*_ values to those of the Guinier analyses, demonstrating the self-consistency of the data (open symbols, Fig 5). The maximum lengths of IgG4 were determined from the *r* value when the *P(r)* curve intersected zero, and was 15 nm for the four X-ray samples (Fig 6*A*). The maximum lengths were similar but slightly increased to 16 nm for the four neutron samples (Fig 6*B*).

The maxima of the *P(r*) curves corresponded to the most frequent distances within the IgG4 structures, and monitored its solution structures. As seen previously for antibodies, two peaks, *M1* and *M2*, were visible. Their values were measured directly from the *P(r)* maxima (Fig 6), although the *M2* values were less precise for reason of sometimes appearing as a shoulder in the *P(r)* curves. Peak *M1* was assigned to the Fab and Fc subunits which have shorter distances, and should be invariant because these subunits should not change shape after glycan removal. Peak *M2* was assigned to the relative separation of the Fab and Fc subunits, and monitored changes in this separation (Figs 1 and 6). Direct measurements of the *M1* and *M2* positions showed no differences in their positions before and after deglycosylation (Fig 6*C* and 6*D*). By X-rays, the *M1* and *M2* peaks occurred at 4.54 ± 0.02 nm and 7.29 ± 0.07 nm respectively for glycosylated IgG4 and 4.53 ± 0.01 nm and 7.27 ± 0.05 nm for deglycosylated IgG4. By neutrons, the *M1* and *M2* peaks occurred at 4.38 ± 0.08 nm and 7.23 ± 0.20 nm respectively for glycosylated IgG4 and 4.29 ± 0.03 nm and 7.17 ± 0.12 nm respectively for deglycosylated IgG4. The X-ray and neutron analyses for IgG4 were thus consistent with each other.

### Scattering modelling of glycosylated and deglycosylated IgG4

Modelling simulations of IgG4 before and after glycan removal assessed potential conformational differences between the two structures. The scattering curves were modelled using high-resolution crystal structures for the human Fab and Fc subunits (Materials and Methods). The Fab sequence in its crystal structure was converted using Modeller into that for IgG4 A33 (S1*A–S1D* Fig in S1 File). The Fab and Fc subunits were connected with PyMOL using the sequence ^216^ESKYGPPCPPCPAPEFLGGP^235^ which included the 12-residue hinge (S1*E* Fig in S1 File). Where needed, two complete biantennary glycans were added to the Fc subunit (Fig 1*B*), but not including core fucosylation. This initial structure was energy minimised.

**Fig 7:**
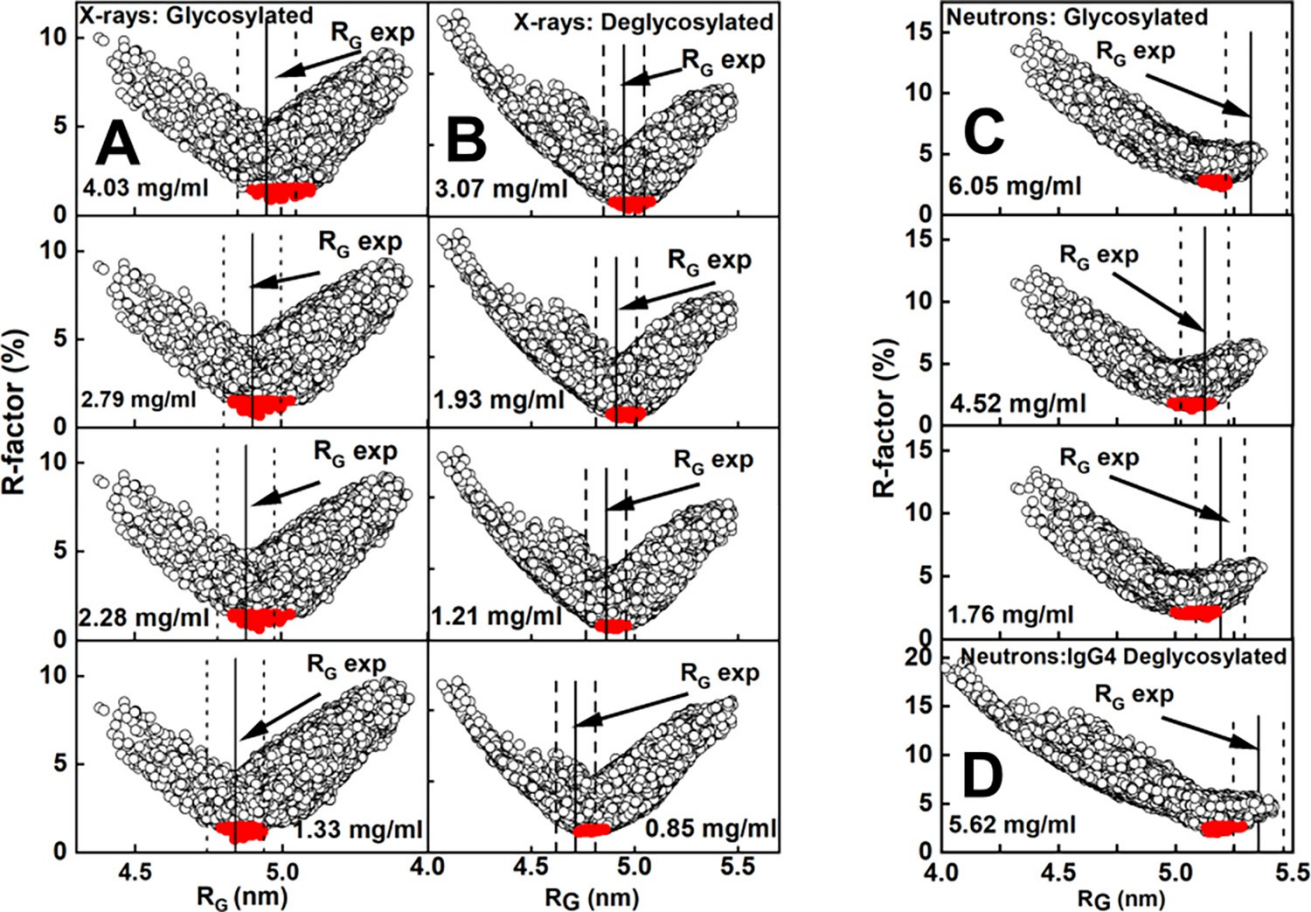
Atomistic modelling of IgG4. The *R-*factor (Materials and Methods) fit parameters for 111,382 models for glycosylated IgG4 and 117,135 models for TP10 deglycosylated IgG4 are shown as circles against the experimental curves. The top 100 best-fit models (red circles) showed the lowest goodness-of-fit *R*-factors. The experimental *R*_*G*_ value is represented by a solid vertical line and the dashed vertical lines represent the ± 2% upper and lower error boundaries. (*A*,*B*) X-ray *R*-factor fits for glycosylated and deglycosylated IgG4 at four concentrations each are shown. (*C*,*D*) Neutron *R*-factor fits for glycosylated and deglycosylated IgG4 at three and one concentrations each are shown.

Libraries of IgG4 models were created by assigning three three-residue segments at the start and end of the two IgG4 hinges to be variable in their torsion angles (green circles, Fig 1B). Each library represented relative movements of the two Fab and one Fc structures between each other. For glycosylated IgG4, eight Monte Carlo simulations gave 800,000 models, of which 111,382 were acceptable for reason of the absence of steric clashes in the IgG4 model. For deglycosylated IgG4, six simulations gave 600,000 models that resulted in 117,135 sterically acceptable structures. As a control of any systematic trends in the curve fits, these fits utilised four experimental X-ray and up to three neutron curves in multiple concentrations for the glycosylated and TP10 deglycosylated IgG4 samples (Fig 7). This comparison of the four experimental X-ray curves at 0.85–4.03 mg/ml with the 111,382 and 117,135 trial curves gave clear chevron-shaped minima in graphs of the goodness-of-fit *R*-factor vs *R*_*G*_ distribution (Fig 7*A* and 7*B*). The experimental *R*_*G*_ values were close to these minima as desired, although the minima were sometimes skewed when encompassing the +2% upper boundary of these *R*_*G*_ values (Fig 7*A*). The existence of minima showed that sufficient X-ray models had been tested in order to give good scattering curve fits. The 100 best-fit scattering models were selected as those with the lowest *R*-factors with values between 0.65–1.57% for the glycosylated models and 0.53–1.62% for the TP10 deglycosylated models (S2 Table in S1 File) (red, Fig 7). Similar chevron-shaped minima were seen for the neutron fits (Fig 7*C* and 7*D*), although the reduced length of the right-hand arm of the chevon is attributed to the consequence of the near-invisible hydration shell on the IgG4 neutron data.

**Fig 8:**
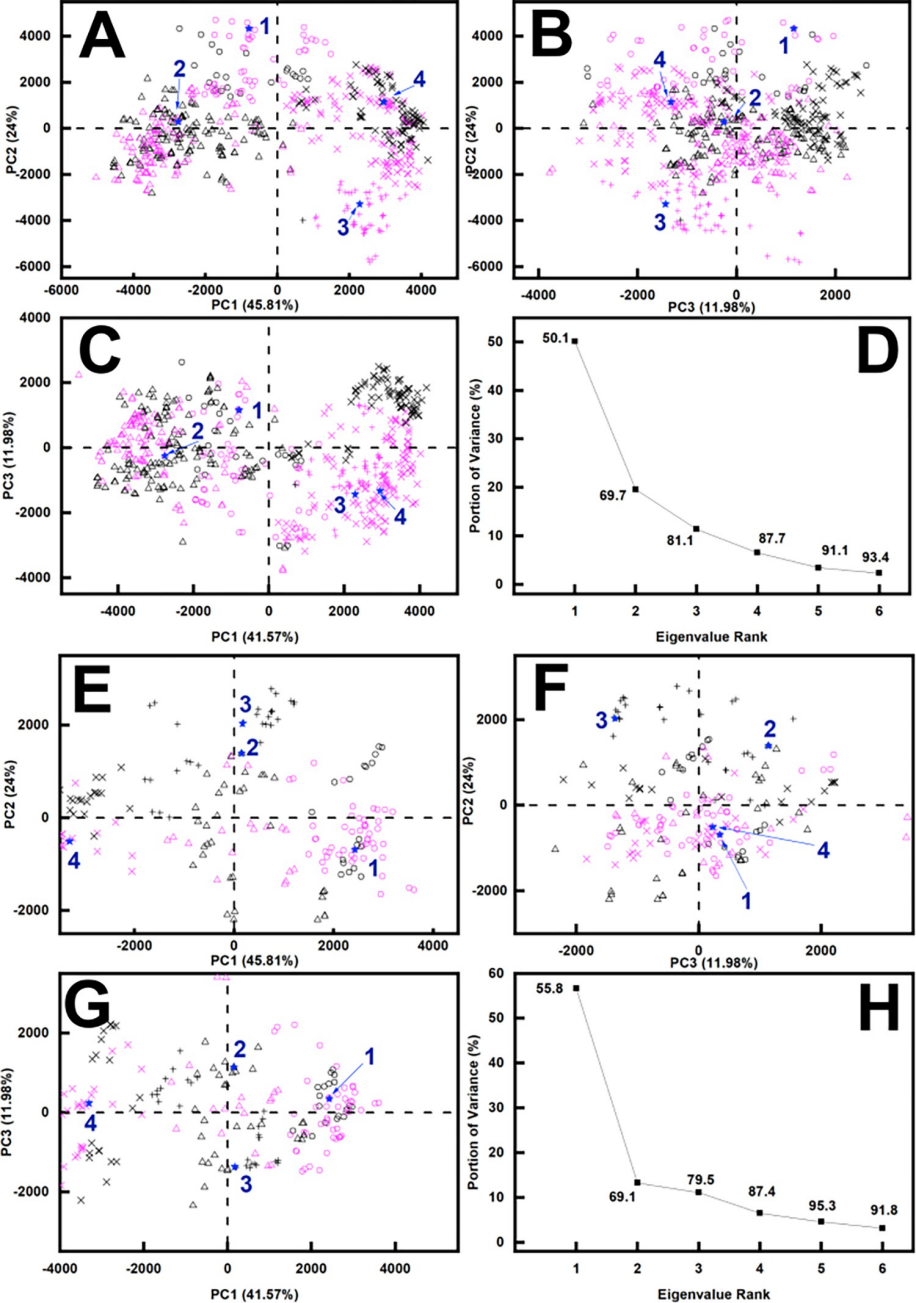
Principal component analysis of the best-fit IgG4 models. Glycosylated best-fit structures are represented in black, and TP10 deglycosylated best-fit structures are represented in magenta. The PCA groups 1, 2, 3 and 4 are represented by ○, Δ, +, and × in that order, and the centroid model for each group is represented by large numbers (blue) and a ★. (A-D) The eight sets of 100 X-ray best-fit models for glycosylated and deglycosylated IgG4 were grouped into four PCA groups as shown in *A*, *B* and *C* of PC2 vs PC1, PC3 vs PC2 and PC3 vs PC1. PC1, PC2 and PC3 are the first three principal components of the analysis. *D*, The first three eigenvalues PC1, PC2 and PC3 captured 81.1% of the variance in the 800 models. (E-H) The two sets of 100 neutron best-fit models for glycosylated and TP10 deglycosylated IgG4 were grouped into four PCA groups as shown in *E*, *F* and *G* of PC2 vs PC1, PC3 vs PC2 and PC3 vs PC1. *H*, The first three eigenvalues PC1, PC2 and PC3 captured 79.5% of the variance in the 200 models.

**Fig 9:**
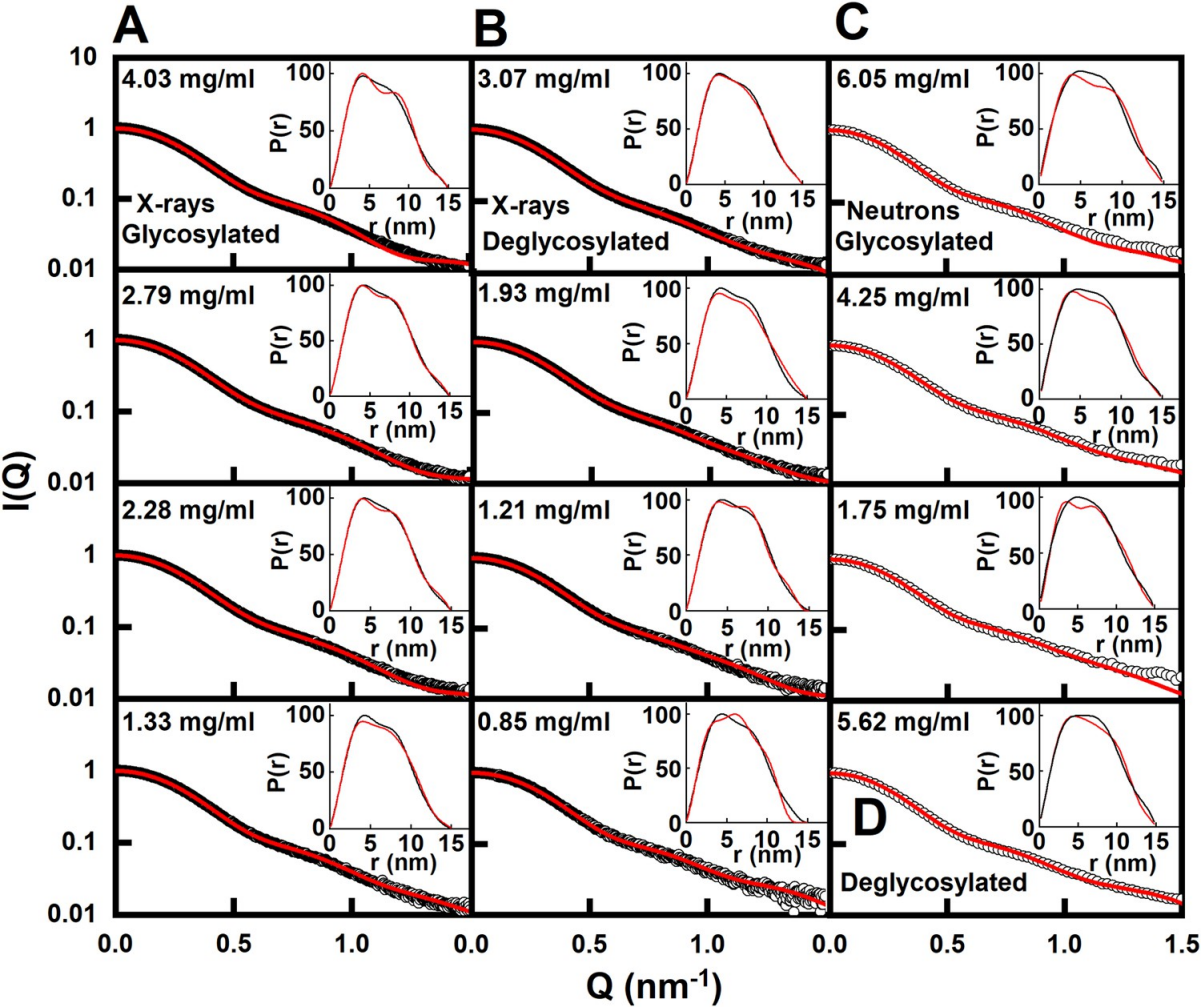
Fits of the best-fit models to the experimental IgG4 scattering data. The individual experimental *I(Q)* curves are denoted by black circles and normalised to *I(0)* = 1. The best-fit modelled curves are denoted by red lines. Note the log scale on the vertical axis. The *P(r)* curves are shown as insets at the top right. The fits are provided in Supplemental Materials. (*A*) Glycosylated and (*B*) TP10 deglycosylated IgG4 X-ray scattering curve fits for four concentrations each. For the four X-ray fits in *A*, the glycosylated IgG4 models were taken from PCA group 1 (2.79 mg/ml, 2.28 mg/ml) and group 2 (4.03 mg/ml and 1.33 mg/ml) in that order (S2 Table in S1 File). In *B*, the deglycosylated IgG4 models corresponded to PCA group 2 (0.85 mg/ml) and group 4 (3.07 mg/ml, 1.93 mg/ml and 1.21 mg/ml in that order). (*C*) Glycosylated and (*D*) TP10 deglycosylated IgG4 neutron fits are shown for three and one concentrations respectively. The glycosylated IgG4 models corresponded to PCA group 2 (4.25 mg/ml) (S3 Table in S1 File). The deglycosylated IgG4 models corresponded to PCA group 1 (5.62 mg/ml). The X-ray experimental curves correspond to glycosylated IgG4 at 4.03 mg/ml and deglycosylated IgG4 at 3.07 mg/ml.

To assess the two sets of best-fit IgG4 structures, principal component analyses (PCA) were carried out [18, 55, 58]. PCA evaluates the correlated motions of amino acid residues to be linearly uncorrelated variables, each being a principal component [55, 58]. A covariance matrix of the atomic coordinates of the frames in the selected structure set was used to extract these “essential motions”. The matrix eigenvectors have an associated eigenvalue that describes the clustering of the models based on their molecular coordinates (or variance). The PCA analyses accounted for about 80% of the variance (Fig 8*D*). To avoid bias in comparing the glycosylated and deglycosylated models, the glycans chains were removed from the glycosylated models-. Interestingly, despite the lack of change in the Guinier or *P(r)* analyses, PCA indicated differences between the two sets of best-fit X-ray IgG4 models (Fig 8*A*–*8C*; S2 Table in S1 File). The analyses showed that the two distributions were each optimally clustered into four distinct Groups 1–4, with some overlap between the glycosylated and deglycosylated ones. Thus PCA Group 2 contained most of the glycosylated models (black), while PCA Groups 1 and 3 contained mostly the deglycosylated models (magenta), and PCA Group 4 contained both the glycosylated and deglycosylated models. The PCA analyses confirmed the validity of the X-ray curve fits (Fig 9*A* and 9*B*), especially the agreement of the experimental *P(r)* curves with the theoretical double peaks in the *P(r)* curves.

The neutron modelling gave this same fit outcome, hence confirming the reproducibility of the modelling analyses. The same 111,382 and 117,135 modelled curves were compared with the neutron curves at 1.75–6.05 mg/ml to give clear minima with 100 best-fit models in each of the *R*-factor vs *R*_*G*_ distributions (Fig 7*C* and 7*D*). Because different sets of scattering data were involved that reflect non-hydrated and hydrated structures, the neutron fits gave a different chevron-shaped outcome from the X-ray fits. The minima in two of the plots encompassed the experimental *R*_*G*_ values, while the minima in the other two plots were skewed to encompass the -2% upper boundary of these *R*_*G*_ values. For the same reason, the four PCA groups do not map 1:1 between the X-ray and neutron fits. The PCA also indicated differences following deglycosylation (Fig 8*E*–8*H*; S3 Table in S1 File). The distributions of the best-fit neutron models (4.52 mg/ml in Fig 7*C*; Fig 7*D*) were contained as four PCA groups again. PCA Groups 2 and 3 contained mostly the glycosylated models (black) while PCA Groups 1 and 4 contained mostly the TP10 deglycosylated models (magenta). The presence and near-absence of the hydration shells in the X-ray and neutron fits respectively revealed the same outcome of conformational differences between the two forms of IgG4 (Fig 9*C* and 9*D*).

**Fig 10:**
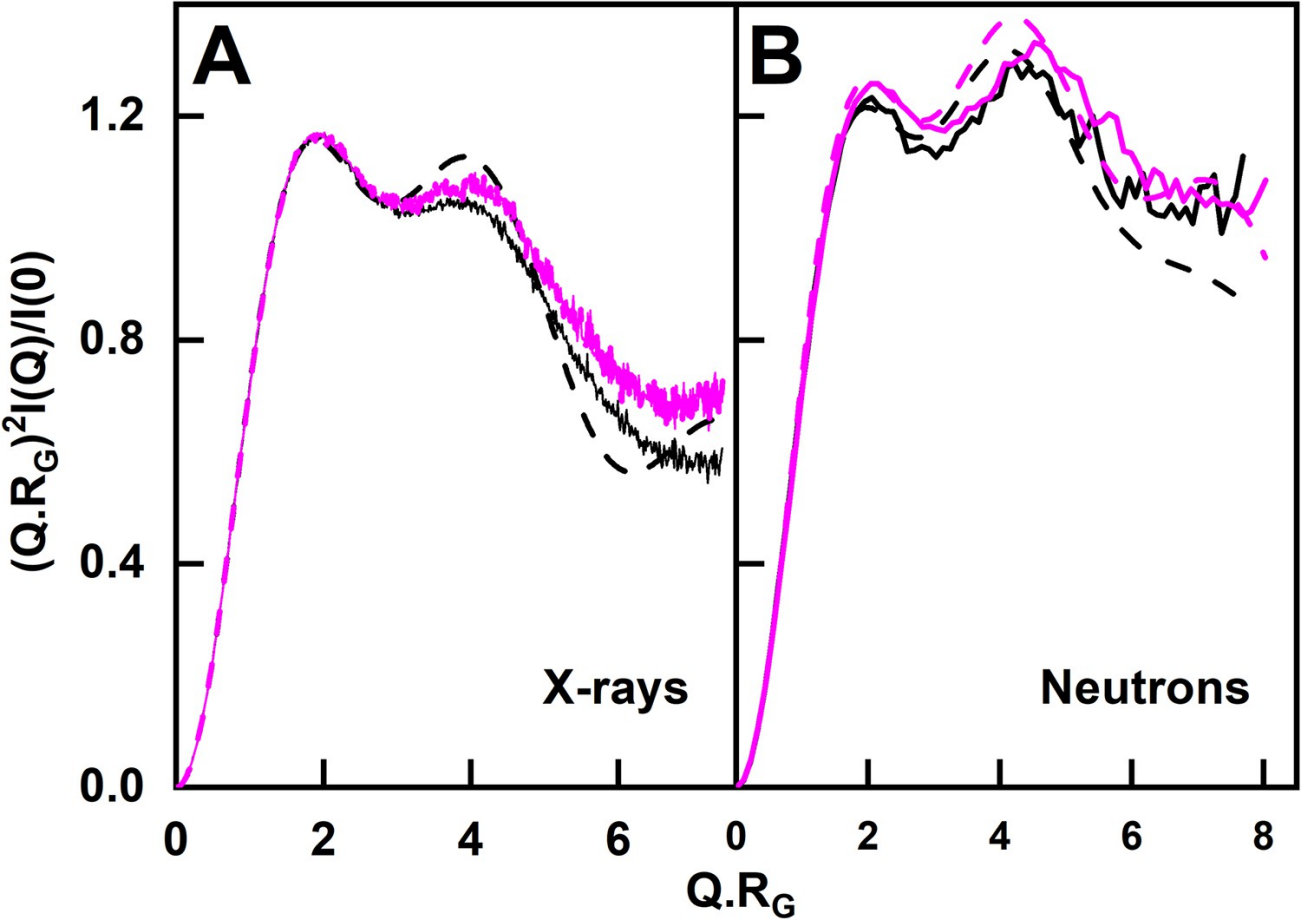
Normalised Kratky plots for the experimental and modelled IgG4 scattering curves. (*A*) X-ray data (solid lines) and modelled fits (dashed lines) are shown for glycosylated IgG4 at 4.03 mg/ml (black) and deglycosylated IgG4 at 3.07 mg/ml (magenta). (*B*) Neutron data (solid lines) and modelled fits (dashed lines) are shown for glycosylated IgG4 at 4.52 mg/ml (black) and for deglycosylated IgG4 at 5.62 mg/ml (magenta).

Further insights on the IgG4 structures were revealed from wide-angle Kratky plots of *(Q*.*R*_*G*_*)*^*2*^.*I(Q)/I(0) vs Q*.*R*_*G*_ for both the experimental and modelled curves. Kratky plots indicate whether a protein has a compact globular structure or possesses disordered regions [59]. Two peaks were seen in the experimental and modelled curves. For the X-ray data for glycosylated IgG4, the peaks occurred at *Q*.*R*_*G*_ values of 2.02 and 3.95, in good agreement with the modelling that showed peaks at 1.89 and 3.92 (Fig 10*A*). For deglycosylated IgG4, the X-ray peaks were similar at 2.01 and 4.06, in good agreement with the modelled peaks at 2.01 and 4.18. It was noteworthy that the X-ray peak at 4.06 showed higher intensities for deglycosylated IgG4 (magenta) than glycosylated IgG4 (black), indicating a slightly increased disorder after deglycosylation. For glycosylated IgG4, the neutron peaks occurred at 2.06 and 4.22, which were similar to the modelled values at 1.95 and 4.11 (Fig 10*B*). For deglycosylated IgG4, the neutron peaks were at 2.15 and 4.52, in accord with the modelled peaks at 2.03 and 4.18. Here, the second peak showed increased intensities for both experimental and modelled deglycosylated IgG4 (magenta) compared to glycosylated IgG4 (black). Hence the neutron plots also suggested more degrees of freedom in IgG4 to move after deglycosylation. These observations may explain what is observed in the PCA description of differences after deglycosylation.

The output of the scattering modelling was used to calculate the *s*^*0*^_*20*,*w*_ values for the best-fit 100 glycosylated and deglycosylated structures for each X-ray concentration (Figs 7 and 9) using HullRad [56]. The range of *s*^*0*^_*20*,*w*_ values was 6.68–6.99 S for glycosylated IgG4 and 6.39–6.70 S for deglycosylated IgG4 (S2 Table in S1 File). Given that these comparisons generally agree within ± 0.21 S for similar macromolecules [60], the modelled values are in good accord with the observed *s*^*0*^_*20*,*w*_ values of 6.54–6.55 S for glycosylated IgG4 and 6.03–6.33 S for deglycosylated IgG4 (S1 Table in S1 File). These *s*^*0*^_*20*,*w*_ agreements support the scattering modelling.

## Discussion

### Changes in IgG4 upon deglycosylation

The structural role of the Fc glycans of IgG4 has been rationalised by this study. There are moves in industry to adopt non-glycosylated monoclonal antibodies, and one of the drivers is the potential for alternative manufacturing platforms including bacterial and also cell free systems [61]. Knowledge of the conformations of glycosylated and deglycosylated IgG4 will help inform this trend. Compared to earlier crystallographic studies on only IgG4-Fc, our joint X-ray/neutron-AUC-MC approach has analysed full-length IgG4 for the structural changes accompanying glycan removal (Fig *1B*). The AUC data showed that deglycosylated IgG4 was monomeric and that the *s*^*0*^_*20*,*w*_ values decreased with the decrease in molecular mass of IgG4 after glycan removal. The X-ray and neutron data collection on IgG4 before and after glycan removal gave a full set of Guinier parameters, and their corresponding distance distribution curves *P(r)*. Interestingly no differences in these were observed between glycosylated and deglycosylated IgG4. This is in contrast to IgG1 where clear differences in the *R*_*XS-1*_ and *M2* parameters reflected increased Fab-Fc separations after deglycosylation [18]. These differences are attributed to the longer length of the hinge in IgG1 compared to IgG4. Nonetheless, differences between the two forms of IgG4 were only seen in the different PCA analyses of the two forms and the slightly greater disorder seen in the Kratky plots for IgG4 following its deglycosylation (Fig 10), potentially indicating more conformational sampling, and suggesting that the structural changes are minor.

**Fig 11:**
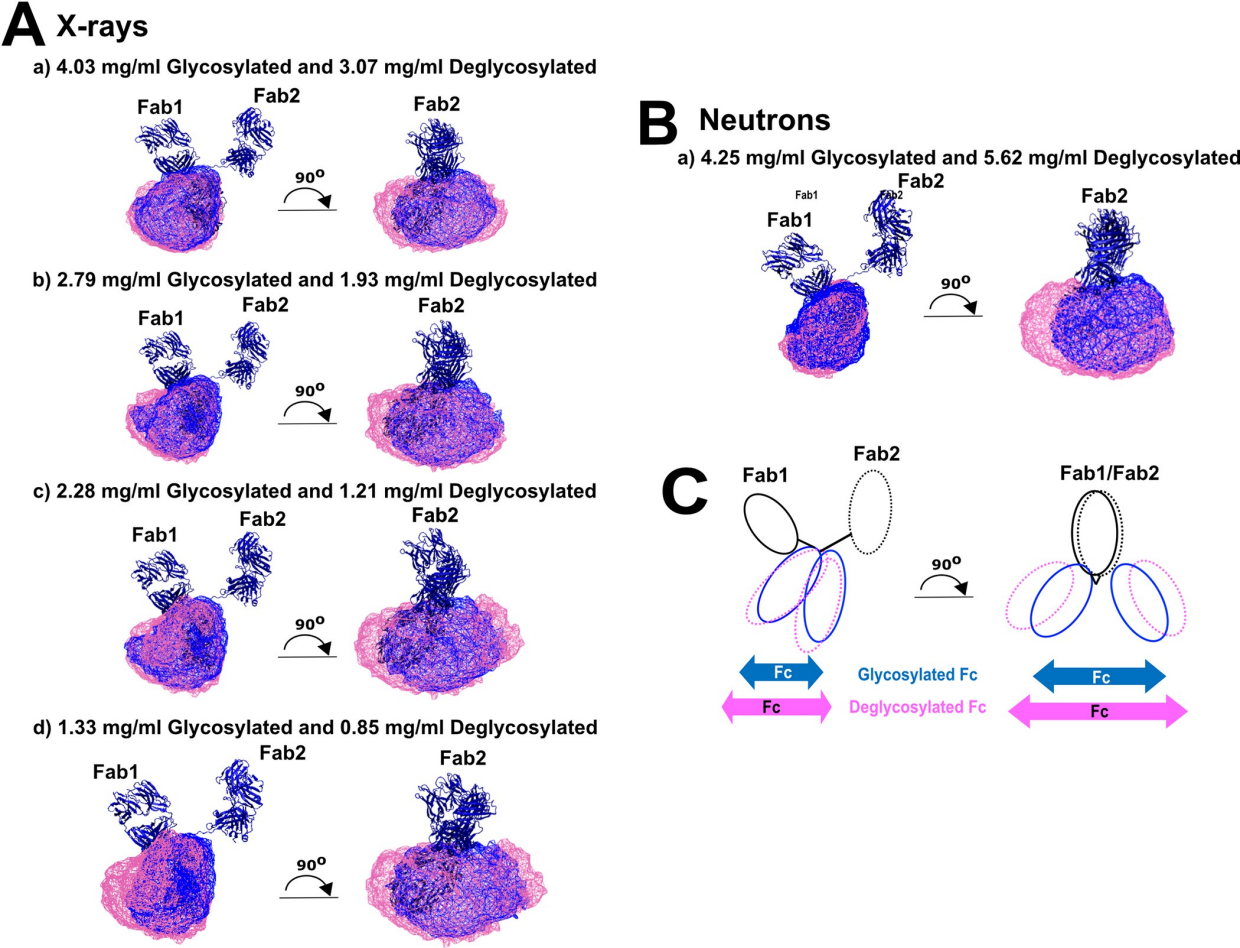
Representative best-fit X-ray and neutron structures. The blue ribbons show the backbone of the starting glycosylated and deglycosylated IgG4 protein models. The Fab subunits of each set of 100 best-fit models were superimposed with these starting structures, in order to highlight movements in the Fc subunit. The blue and magenta wireframes indicate the space taken by the glycosylated and deglycosylated Fc subunits, respectively, in the 100 best-fit structures from (*A*) four X-ray and (*B*) one neutron analyses. In all cases, the magenta wireframes for deglycosylated IgG4 take more space compared to the blue wireframes for glycosylated IgG4. (*C*) The cartoon representation was based on part (c) of (*A*) and highlighted the greater range of Fc conformations (magenta) after deglycosylation in comparison to that for glycosylated IgG4 (blue).

The scattering modelling using Monte Carlo simulations of 111,382–117,135 stereochemically-correct IgG4 conformations clarified the structural importance of the C_H_2 glycans in IgG4. The PCA analyses of the best-fit models showed that, despite minor similarities between the glycosylated and deglycosylated models, the two sets of IgG4 PCA structures were clustered into distinct groups (Fig 8). Using wireframe representations for clarity, the four sets of 100 best-fit X-ray models show that the Fc subunit of glycosylated models were slightly more compactly superimposed (blue, Fig 11*A*) when compared to the deglycosylated model (magenta, Fig 11*A*). This indicates that glycosylated IgG4 can access fewer conformational states. This result is simplified in the cartoon of Fig 11*C*. Compared to IgG1, the shorter length of the IgG4 hinge results in a reduced level of conformational flexibility. This would account for the differences observed in the IgG1 but not the IgG4 scattering analyses. For IgG1, the *R*_*XS-1*_ and *M2* values had shifted, but this did not occur for IgG4. The best-fit IgG4 neutron models also followed this interpretation (Fig 11*B*). Overall the molecular simulations accounted for small structural differences seen before and after deglycosylation in IgG4, but did not reveal larger changes comparable to those in IgG1.

### Functional significance of glycan removal

In relation to function, the main advantage of our study was the analysis of the full-length functional IgG4 structure in solution. Earlier studies of only the IgG1-Fc and IgG4-Fc subunits indicated that the Fc glycans make no or very little contact with the FcγR receptors that mediate the effector function of IgG antibodies [62]. Functional studies of these IgG-FcγR complexes are limited by the availability of crystal structures for only IgG1-Fc complexes, but not for IgG4-Fc in complex with the FcγRs. Functional studies of deglycosylated IgG4 demonstrated abrogated binding to FcγRIIIa, which implicated a role for the glycans to assist in the FcγR interaction [11]. The lower binding affinity of IgG4 can start to be rationalised by our finding in the present study that the deglycosylated IgG4-Fc subunit is more conformationally labile than the seemingly more restricted glycosylated IgG4-Fc subunit [18]. In support of this outcome, crystal structures for deglycosylated Fc showed that its FG loop, which is vital in FcγR and C1q binding, can adopt two distinct conformations that would reduce their binding interactions [62]. As a different perspective altogether, one previous structural study of the full-length IgG4 antibody suggested that the IgG4 glycans may not reside in the internal cavity of the Fc subunit, as shown in Fig 1*B*, but are solvent exposed [12]. That study attributed this outcome to the shorter IgG4 hinge that forced the C_H_2 domains into an unorthodox conformation that differed from the available IgG4-Fc crystal structures [14–16]. Such a C_H_2 domain rearrangement may itself reduce IgG4 function because the contact residues for FcγR and C1q binding have been displaced, however this appears unlikely.

### Utility of modelling to study antibodies

Fitting atomistic structures to the IgG4 scattering curves significantly improves the utility of solution scattering. We have now made three such studies, each progressing the capabilities of the method. Our first IgG4 modelling analysis in 2014 used SCT/SCTPL software based on small spheres [39] to look at 20,000 trial IgG4 models created using crystal structures for the Fab and Fc subunits and a randomised hinge [57]. The resulting good fits showed that the IgG4-Fab subunits restricted access to the Fc subunit, this explaining the inability of IgG4 to activate complement. Asymmetric IgG4 structures were determined that agreed with crystal structures of full length human IgG4 [12, 13]. Our second IgG4 modelling using SASSIE employed fits based on 190,437 Monte Carlo models [35]. This gave best-fit R-factors of 3%. This gave symmetric and asymmetric IgG4 solution structures, and docking simulations of the IgG4-FcγRI interaction showed greater steric clashes, when compared to IgG1. This outcome explained the observed lower binding affinity of the IgG4-FcγRI interaction compared to that of IgG1. One difference between the two studies is that SCT/SCTPL explicitly incorporated hydration shells in a coarse-grained approach [38], while hydration shells were not included in the SASSIE modelling because this is computationally expensive [54] Nonetheless both IgG4 modelling studies resulted in similar asymmetric IgG4 solution structures. The present third modelling used X-ray and neutron curves measured out to *Q* values of 1.5 nm^-1^ to give fits with improved *R*-factors of 1.50% or less (S2 Table in S1 File). The availability of improved signal-noise ratios enabled the study of even smaller potential structural perturbations such as the effect of deglycosylation in the IgG4 structure.

The Supporting information presents the sequence alignment used to generate the IgG4 molecular models. The Tables summarise the Guinier parameters for both forms of IgG4, and the two sets of modelling fits in light and heavy water. The experimentally observed scattering curves and the structural coordinates of our final best-fit models are available as a zip file. The single best-fit IgG4 model was deposited in the SASBDB database with reference SASDP89 (https://www.sasbdb.org/).

## Supporting information

S1 File The supporting information presents the sequence alignment of human IgG4 with other known human IgG4 sequences, and three tables that present a summary of the experimental X-ray and neutron scattering data for glycosylated and deglycosylated IgG4, a summary of the outcome of the X-ray scattering curve modelling, and a summary of the neutron X-ray scattering curve modelling. (DOCX)

S2 File The zip file presents the experimentally observed scattering curves and the structural coordinates of our final best-fit models. (ZIP)

S3 File The original uncropped and unadjusted image is provided for the right-hand image of Fig 2B in the main manuscript. The original uncropped and unadjusted image is provided for the left-hand image of Fig 2B in the main manuscript.
(ZIP)

## Abbreviations

AUC: analytical ultracentrifugation
CCP-SAS: collaborative computational project for small angle scattering
IgG4: immunoglobulin G subclass 4
MC: Monte Carlo
MD: molecular dynamics
PDB: protein databank
PCA: principal component analyses; *R*_*G*_, radius of gyration
SANS: small angle neutron scattering
SAXS: small angle X-ray scattering
