# Supplementary material for: Using atomistic solution scattering modelling to elucidate the role of the Fc glycans in human IgG4
Source: PLoS One. 2024 Apr 1;19(4):e0300964. doi: 10.1371/journal.pone.0300964 (PMC10984405; doi:10.1371/journal.pone.0300964)
Supplement: S1 File — (DOCX) [file pone.0300964.s001.docx]

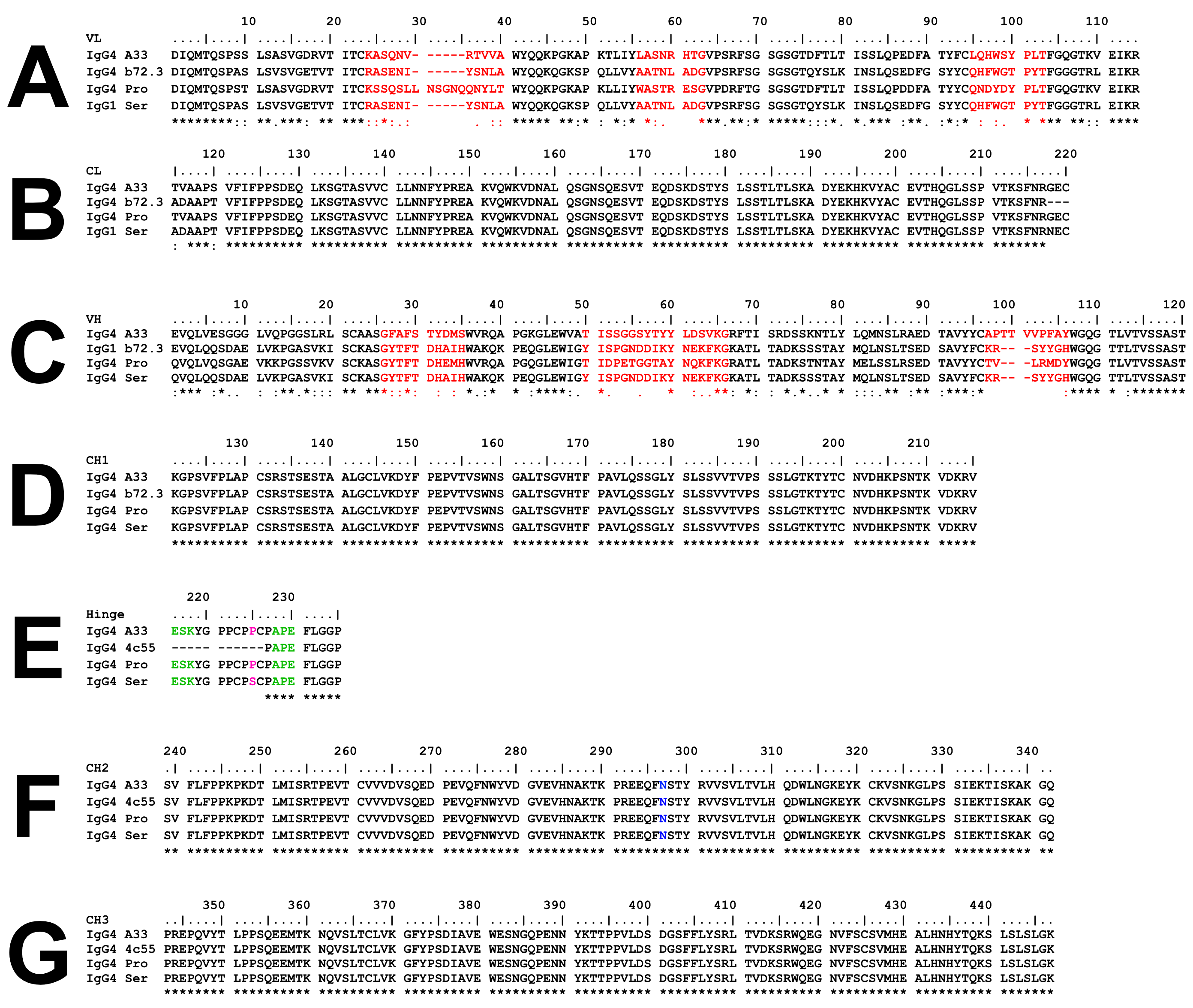


**SUPPLEMENTARY FIGURE S1. Sequence alignment of human IgG4.** (*A-G*) The IgG4 A33 sequence was provided by UCB Pharma. The IgG4 b72.3 and Fc sequences were taken from their crystal structures (PDB ID: 1BBJ, 4C55). *A, B*, the V_L_ and C_L_ domains; *C-D*, the V_H_ and C_H_1 domains; *E*, the hinge with green denoting the TAMC-varied tripeptides, and pink identifying the S225P mutation; *F, G*, the C_H_2 and C_H_3 domains, with blue indicating Asn-297. The commonly-used EU sequence numbering for the constant domains was used and red denotes the complementarity-determining region (CDR) sequences. Beneath the alignments, consensus symbols indicated the degree of conservation, where (*) indicates full conservation, (:) indicates conservation between groups of strongly similar properties based on the Gonnet PAM 250 matrix, (.) indicates conservation between groups of weakly similar properties, and a space indicates no conservation.

.

**SUPPLEMENTARY TABLE S1.** Summary of experimental SAXS and SANS data for glycosylated and deglycosylated IgG4. For SAXS only a selection of four concentrations are shown for each sample.

|  | Concentration | R*_G_* | R*_XS-1_* | R*_XS-2_* | *L* |
| --- | --- | --- | --- | --- | --- |
|  | (mg/ml) | (nm) | (nm) | (nm) | (nm) |
| SAXS data |  |  |  |  |  |
| IgG4 Glycosylated | 4.03 | 4.94 ± 0.27 | 2.51 ± 0.16 | 1.42 ± 0.12 | 15 |
|  | 2.79 | 4.89 ± 0.29 | 2.50 ± 0.20 | 1.41 ± 0.14 | 15 |
|  | 2.28 | 4.90 ± 0.29 | 2.50 ± 0.18 | 1.39 ± 0.13 | 15 |
|  | 1.33 | 4.82 ± 0.35 | 2.50 ± 0.19 | 1.40 ± 0.16 | 15 |
| IgG4 TP1 | 4.70 | 5.00 ± 0.27 | 2.51 ± 0.16 | 1.41 ± 0.11 | 15 |
|  | 4.20 | 4.98 ± 0.29 | 2.52 ± 0.16 | 1.40 ± 0.12 | 15 |
|  | 1.87 | 4.88 ± 0.31 | 2.51 ± 0.17 | 1.41 ± 0.14 | 15 |
|  | 1.63 | 4.88 ± 0.32 | 2.50 ± 0.19 | 1.39 ± 0.15 | 15 |
| IgG4 TP6 | 3.10 | 4.97 ± 0.29 | 2.52 ± 0.16 | 1.40 ± 0.13 | 15 |
|  | 2.24 | 4.94 ± 0.32 | 2.51 ± 0.16 | 1.40 ± 0.14 | 15 |
|  | 1.79 | 4.91 ± 0.34 | 2.51 ± 0.18 | 1.40 ± 0.14 | 15 |
|  | 1.06 | 4.85 ± 0.40 | 2.49 ± 0.20 | 1.38 ± 0.17 | 15 |
| IgG4 TP10 | 3.07 | 4.95 ± 0.29 | 2.51 ± 0.16 | 1.38 ± 0.13 | 15 |
|  | 1.93 | 4.91 ± 0.30 | 2.50 ± 0.19 | 1.39 ± 0.15 | 15 |
|  | 1.21 | 4.86 ± 0.36 | 2.49 ± 0.20 | 1.36 ± 0.17 | 15 |
|  | 0.85 | 4.72 ± 0.41 | 2.44 ± 0.23 | 1.33 ± 0.18 | 15 |
| SANS data |  |  |  |  |  |
| IgG4 Glycosylated | 6.05 | 5.32 ± 0.99 | 2.41 ± 0.29 | 1.15 ± 0.22 | 16 |
|  | 4.52 | 5.12 ± 0.77 | 2.47 ± 0.31 | 1.20 ± 0.24 | 16 |
|  | 1.76 | 5.19 ± 0.88 | 2.43 ± 0.30 | 1.11 ± 0.25 | 16 |
| IgG4 TP1 | 1.93 | 5.19 ± 0.78 | 2.43 ± 0.39 | 1.13 ± 0.24 | 16 |
|  | 0.71 | 5.32 ± 0.63 | 2.48 ± 0.35 | 1.22 ± 0.25 | 16 |
| IgG4 TP6 | 2.38 | 5.12 ± 0.75 | 2.46 ± 0.26 | 1.21 ± 0.22 | 16 |
| IgG4 TP10 | 5.62 | 5.35 ± 0.79 | 2.50 ± 0.22 | 1.20 ± 0.19 | 16 |

**SUPPLEMENTARY TABLE S2.** Modelling fits for the SAXS and AUC data in light water

| Filter | Models | R*_G_* before minimization | R*_G_* after minimization | R*_XS-1_* | R*_XS-2_* | *L* | R-factor before minimization | R-factor after minimization | *s_20,w_* |
| --- | --- | --- | --- | --- | --- | --- | --- | --- | --- |
|  |  | (nm) | (nm) | (nm) | (nm) | (nm) | (%) | (%) | (S) |
| Library of glycosylated models | 111382 | 4.38-5.43 | NA | NA | NA | NA | 0.65-9.99 | NA | NA |
| Top 100 at 4.03 mg/ml | 100 | 4.90-5.10 | 4.90-5.10 | 2.39-2.58 | 1.25-1.61 | NA | 0.89-1.57 | 0.81-1.55 | 6.68-6.89 |
| Best fit at 4.03 mg/ml | 1 | 4.96 | 4.97 | 2.54 | 1.50 | 15 | 0.89 | 0.86 | 6.73 |
| Top 100 at 2.79 mg/ml | 100 | 4.83-5.03 | 4.83-5.03 | 2.39-2.59 | 1.18-1.67 | NA | 0.69-1.53 | 0.61-1.52 | 6.69-6.91 |
| Best fit at 2.79 mg/ml | 1 | 4.93 | 4.93 | 2.50 | 1.36 | 15 | 0.69 | 0.61 | 6.72 |
| Top 100 at 2.28 mg/ml | 100 | 4.83-5.03 | 4.83-5.03 | 2.39-2.59 | 1.20-1.68 | NA | 0.65-1.47 | 0.59-1.48 | 6.69-6.91 |
| Best fit at 2.28 mg/ml | 1 | 4.93 | 4.93 | 2.50 | 1.35 | 15 | 0.65 | 0.59 | 6.72 |
| Top 100 at 1.38 mg/ml | 100 | 4.79-4.93 | 4.79-4.93 | 2.42-2.61 | 1.16-1.61 | NA | 0.75-1.47 | 0.68-1.46 | 6.72-6.99 |
| Best fit at 1.38 mg/ml | 1 | 4.84 | 4.84 | 2.53 | 1.40 | 15 | 0.75 | 0.68 | 6.85 |
| Library of deglycosylated models | 117135 | 4.07-5.47 | NA | NA | NA | NA | 0.53-14.15 | NA | NA |
| Top 100 at 3.07 mg/ml | 100 | 4.90-5.08 | 4.90-5.08 | 2.42-2.61 | 1.19-1.56 | NA | 0.53-1.03 | 0.56-1.07 | 6.39-6.54 |
| Best fit at 3.07 mg/ml | 1 | 5.02 | 5.02 | 2.48 | 1.45 | 15 | 0.53 | 0.57 | 6.47 |
| Top 100 at 1.93 mg/ml | 100 | 4.88-5.03 | 4.88-5.03 | 2.41-2.61 | 1.17-1.59 | NA | 0.66-1.11 | 0.66-1.15 | 6.39-6.59 |
| Best fit at 1.93 mg/ml | 1 | 4.98 | 4.98 | 2.48 | 1.45 | 15 | 0.66 | 0.66 | 6.52 |
| Top 100 at 1.21 mg/ml | 100 | 4.83-4.96 | 4.83-4.96 | 2.43-2.57 | 1.21-1.52 | NA | 0.81-1.07 | 0.79-1.10 | 6.44-6.66 |
| Best fit at 1.21 mg/ml | 1 | 4.92 | 4.91 | 2.49 | 1.31 | 15 | 0.81 | 0.79 | 6.49 |
| Top 100 at 0.85 mg/ml | 100 | 4.73-4.86 | 4.73-4.86 | 2.41-2.56 | 1.15-1.47 | NA | 1.42-1.62 | 1.41-1.67 | 6.50-6.70 |
| Best fit at 0.85 mg/ml | 1 | 4.74 | 4.73 | 2.51 | 1.30 | 15 | 1.42 | 1.41 | 6.68 |
| PCA Group 1 | 101 | 4.77-5.10 | 4.77-5.10 | 2.40-2.58 | 1.16-1.56 | NA | 0.65-1.62 | 0.59-1.65 | 6.41-6.84 |
| Glycosylated | 41 | 4.84-5.10 | 4.84-5.10 | 2.40-2.58 | 1.16-1.52 | NA | 0.65-1.57 | 0.59-1.51 | 6.69-6.84 |
| Deglycosylated | 60 | 4.77-5.08 | 4.77-5.08 | 2.42-2.58 | 1.21-1.56 | NA | 0.70-1.62 | 0.71-165 | 6.41-6.69 |
| Centroid | 1 | 4.91 | 4.91 | 2.55 | 1.40 | 15 | 1.10 | 1.03 | 6.54 |
| PCA Group 2 | 342 | 4.73-5.08 | 4.73-5.08 | 2.37-2.58 | 1.18-1.68 | NA | 0.54-1.61 | 0.56-1.63 | 6.38-6.99 |
| Glycosylated | 218 | 4.82-5.08 | 4.82-5.08 | 2.37-2.58 | 1.18-1.68 | NA | 0.74-1.56 | 0.69-1.52 | 6.68-6.99 |
| Deglycosylated | 124 | 4.73-5.07 | 4.73-5.07 | 2.41-2.56 | 1.21-1.56 | NA | 0.54-1.61 | 0.56-1.63 | 6.41-6.69 |
| Centroid | 1 | 4.80 | 4.80 | 2.43 | 1.38 | 15 | 1.60 | 1.64 | 6.57 |
| PCA Group 3 | 53 | 4.75-5.04 | 4.75-5.04 | 2.41-2.55 | 1.15-1.56 | NA | 0.73-1.62 | 0.71-1.67 | 6.45-6.77 |
| Glycosylated | 1 | 4.97 | 4.97 | 2.54 | 1.53 | NA | 1.24 | 1.20 | 6.77 |
| Deglycosylated | 52 | 4.75-5.04 | 4.75-5.04 | 2.41-2.55 | 1.15-1.56 | NA | 0.73-1.62 | 0.71-1.67 | 6.45-6.70 |
| Centroid | 1 | 4.80 | 4.80 | 2.42 | 1.33 | 15 | 1.60 | 1.63 | 6.68 |
| PCA Group 4 | 304 | 4.77-5.09 | 4.77-5.09 | 2.41-2.61 | 1.17-1.59 | NA | 0.53-1.60 | 0.57-1.60 | 6.39-6.90 |
| Glycosylated | 140 | 4.79-5.09 | 4.79-5.09 | 2.44-2.61 | 1.20-1.56 | NA | 0.88-1.53 | 0.88-1.42 | 6.72-6.90 |
| Deglycosylated | 164 | 4.77-5.06 | 4.77-5.06 | 2.41-2.61 | 1.17-1.59 | NA | 0.53-1.60 | 0.57-1.60 | 6.39-6.68 |
| Centroid | 1 | 4.90 | 4.90 | 2.55 | 1.38 | 15 | 1.03 | 1.00 | 6.50 |

NA, not applicable

**SUPPLEMENTARY TABLE S3.** Modelling fits for the SANS and AUC data in heavy water. NA, not applicable

| Filter | Models | R*_G_* before minimization | R*_G_* after minimization | R*_XS-1_* | R*_XS-2_* | *L* | R-factor before minimization | R-factor after minimization | *s_20,w_* |
| --- | --- | --- | --- | --- | --- | --- | --- | --- | --- |
|  |  | (nm) | (nm) | (nm) | (nm) | (nm) | (%) | (%) | (S) |
| Library of glycosylated models | 111382 | 4.32-5.37 | NA | NA | NA | NA | 0.13-14.85 | NA | NA |
| Top 100 at 6.05 mg/ml | 100 | 5.12-5.22 | 5.12-5.22 | 2.43-2.58 | 1.21-1.51 | NA | 2.30-2.90 | 2.35-2.90 | 6.53-6.67 |
| Best fit at 6.05 mg/ml | 1 | 5.20 | 5.20 | 2.51 | 1.45 | 15 | 2.30 | 2.35 | 6.63 |
| Top 100 at 4.52 mg/ml | 100 | 4.99-5.16 | 4.99-5.16 | 2.40-2.55 | 1.11-1.51 | NA | 1.34-1.85 | 1.36-1.90 | 6.60-6.78 |
| Best fit at 4.52 mg/ml | 1 | 5.07 | 5.07 | 2.46 | 1.36 | 15 | 1.34 | 1.36 | 6.71 |
| Top 100 at 1.75 mg/ml | 100 | 5.00-5.18 | 4.99-5.17 | 2.37-2.56 | 1.07-1.47 | NA | 1.76-2.30 | 1.80-2.33 | 6.59-6.76 |
| Best fit at 1.75 mg/ml | 1 | 5.14 | 5.13 | 2.43 | 1.31 | 15 | 1.76 | 1.80 | 6.73 |
| Library of deglycosylated models | 117135 | 4.02-5.42 | NA | NA | NA | NA | 2.06-19.47 | NA | NA |
| Top 100 at 5.62 mg/ml | 100 | 5.13-5.29 | 5.13-5.28 | 2.48-2.63 | 1.10-1.53 | NA | 2.04-2.77 | 2.02-2.70 | 6.23-6.41 |
| Best fit at 5.62 mg/ml | 1 | 5.18 | 5.17 | 2.54 | 1.41 | 15 | 2.04 | 2.02 | 6.39 |
| PCA Group 1 | 68 | 5.02-5.29 | 5.02-5.28 | 2.42-2.62 | 1.29-1.53 | NA | 1.61-2.72 | 1.59-2.70 | 6.28-6.73 |
| Glycosylated | 18 | 5.02-5.16 | 5.02-5.16 | 2.42-2.51 | 1.32-1.51 | NA | 1.61-1.83 | 1.59-1.87 | 6.60-6.73 |
| Deglycosylated | 50 | 5.16-5.29 | 5.15-5.28 | 2.48-2.62 | 1.29-1.53 | NA | 2.04-2.72 | 2.02-2.70 | 6.28-6.39 |
| Centroid | 1 | 5.19 | 5.18 | 2.53 | 1.42 | 15 | 2.58 | 2.56 | 6.35 |
| PCA Group 2 | 50 | 5.06-5.22 | 5.06-5.21 | 2.42-2.63 | 1.11-1.49 | NA | 1.34-2.70 | 1.36-2.70 | 6.23-6.75 |
| Glycosylated | 32 | 5.06-5.15 | 5.06-5.16 | 2.42-2.52 | 1.28-1.47 | NA | 1.34-1.85 | 1.36-1.90 | 6.63-6.75 |
| Deglycosylated | 18 | 5.14-5.22 | 5.14-5.21 | 2.51-2.63 | 1.11-1.49 | NA | 2.35-2.70 | 2.36-2.70 | 6.23-6.38 |
| Centroid | 1 | 5.09 | 5.09 | 2.49 | 1.39 | 15 | 1.77 | 1.76 | 6.68 |
| PCA Group 3 | 30 | 4.99-5.09 | 4.99-5.10 | 2.40-2.54 | 1.26-1.46 | NA | 1.60-1.85 | 1.52-1.86 | 6.35-6.78 |
| Glycosylated | 30 | 4.99-5.09 | 4.99-5.10 | 2.40-2.54 | 1.26-1.46 | NA | 1.60-1.85 | 1.52-1.86 | 6.65-6.78 |
| Deglycosylated | 0 | NA | NA | NA | NA | NA | NA | NA | NA |
| Centroid | 1 | 5.02 | 5.03 | 2.54 | 1.34 | 15 | 1.82 | 1.8 | 6.72 |
| PCA Group 4 | 52 | 5.03-5.24 | 5.04-5.24 | 2.42-2.63 | 1.10-1.49 | NA | 1.59-2.68 | 1.59-2.65 | 6.28-6.74 |
| Glycosylated | 20 | 5.03-5.13 | 5.04-5.14 | 2.42-2.55 | 1.11-1.43 | NA | 1.59-1.82 | 1.59-1.81 | 6.66-6.74 |
| Deglycosylated | 32 | 5.13-5.24 | 5.13-5.24 | 2.51-2.63 | 1.10-1.49 | NA | 2.09-2.68 | 2.06-2.65 | 6.28-6.41 |
| Centroid | 1 | 5.16 | 5.15 | 2.59 | 1.19 | 15 | 2.10 | 2.06 | 6.65 |
